# Structural Phylogenomics Retrodicts the Origin of the Genetic Code and Uncovers the Evolutionary Impact of Protein Flexibility

**DOI:** 10.1371/journal.pone.0072225

**Published:** 2013-08-21

**Authors:** Gustavo Caetano-Anollés, Minglei Wang, Derek Caetano-Anollés

**Affiliations:** Evolutionary Bioinformatics Laboratory, Department of Crop Sciences, University of Illinois, Urbana, Illinois, United States of America; Institute of Molecular Genetics IMG-CNR, Italy

## Abstract

The genetic code shapes the genetic repository. Its origin has puzzled molecular scientists for over half a century and remains a long-standing mystery. Here we show that the origin of the genetic code is tightly coupled to the history of aminoacyl-tRNA synthetase enzymes and their interactions with tRNA. A timeline of evolutionary appearance of protein domain families derived from a structural census in hundreds of genomes reveals the early emergence of the ‘operational’ RNA code and the late implementation of the standard genetic code. The emergence of codon specificities and amino acid charging involved tight coevolution of aminoacyl-tRNA synthetases and tRNA structures as well as episodes of structural recruitment. Remarkably, amino acid and dipeptide compositions of single-domain proteins appearing before the standard code suggest archaic synthetases with structures homologous to catalytic domains of tyrosyl-tRNA and seryl-tRNA synthetases were capable of peptide bond formation and aminoacylation. Results reveal that genetics arose through coevolutionary interactions between polypeptides and nucleic acid cofactors as an exacting mechanism that favored flexibility and folding of the emergent proteins. These enhancements of phenotypic robustness were likely internalized into the emerging genetic system with the early rise of modern protein structure.

## Introduction

Aminoacyl-tRNA synthetases (aaRSs) are multidomain protein enzymes that attach L-amino acids to their cognate tRNAs with high specificity [1]. The resulting aminoacyl-tRNAs are the substrates of ribosomal protein synthesis. aaRSs define the algorithmic rules of the genetic code in a two-step reaction that correctly pairs amino acids with tRNA isoacceptors and overcomes an enzymatic step that is ∼10^7^ slower than peptide bond formation. The catalytic aminoacylation domain of the enzyme contains an amino acid binding site capable of activating a specific amino acid by condensation with ATP to form aminoacyl-adenylate. This activated molecule then esterifies the 2′ or 3′-hydroxyl group of the ribose in the 3′ end of the acceptor arm of tRNA. The activation process is highly specific and involves proofreading [2], [3]. The aminoacylation site rejects larger amino acids and an editing site (in an editing domain present in about half of aaRSs) generally hydrolyzes those small amino acids that were incorrectly activated. The enzyme must also recognize the nucleic acid triplets of the anticodon arm of tRNA, usually through additional protein domains, the anticodon-binding domains. In eukaryotes, progressive incorporation (accretion) of a number of domains has endowed aaRSs with functions other than those of translation, and the process follows tightly the increases in complexity of the eukaryotic superkingdom [4]. In contrast, little is known about the origin and history of accretion of the domains that provide the crucial aminoacylation, editing and anticodon-binding specificities, and much less about the emergence of the genetic code.

**Figure 1:**
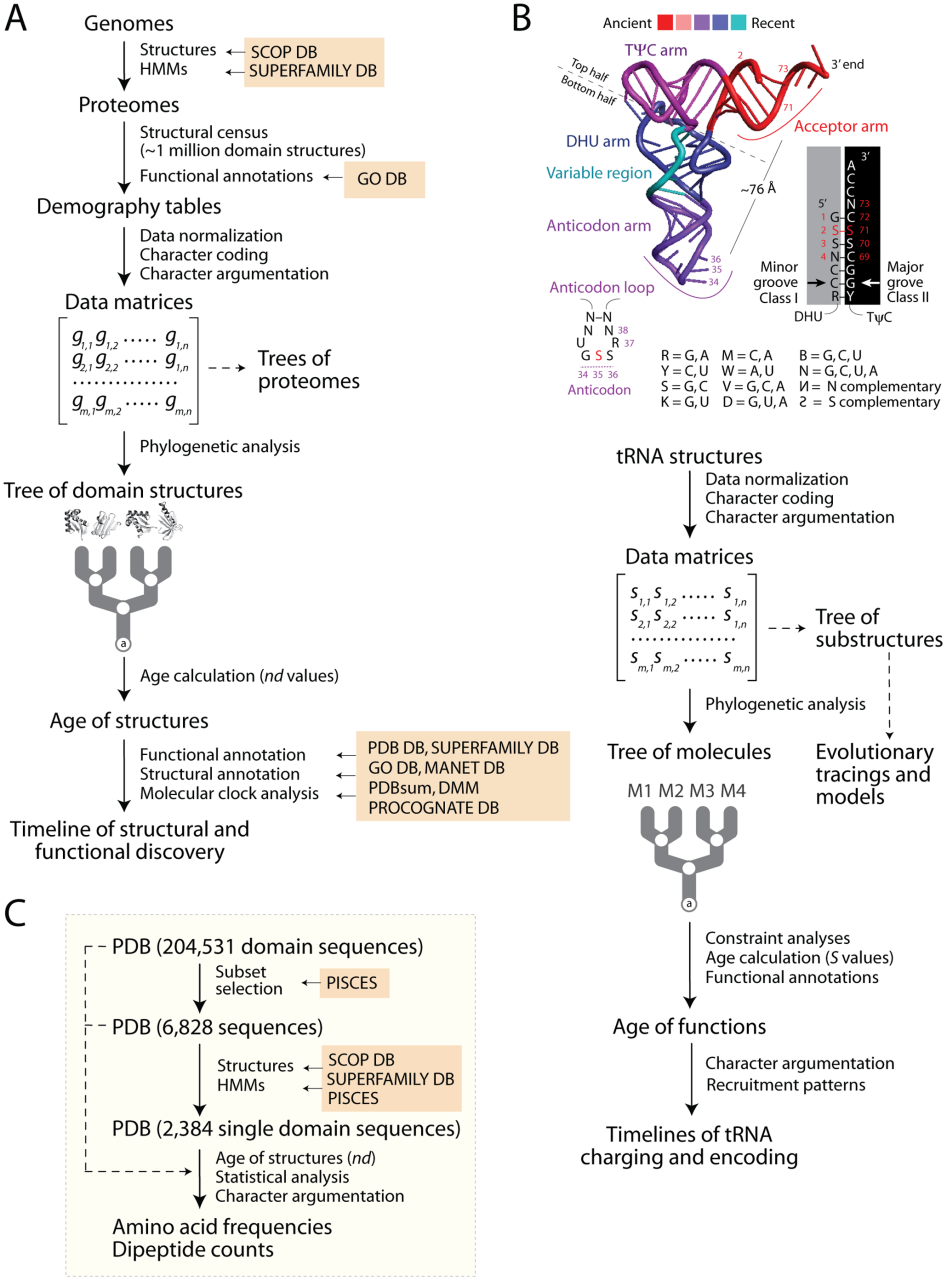
Phylogenomic analyses of protein domains and tRNA structures and functions. A. Flow diagram showing the reconstruction of trees of protein domain structures. A census of domain structures in proteomes of hundreds of completely sequenced organisms is used to compose data matrices, which are then used to build phylogenomic trees describing the evolution of individual protein structures. Elements of the matrix (g) represent genomic abundances of domains in proteomes, defined at different level of classification of domain structure (e.g. SCOP F, FSF, and FF). They are converted into multi-state phylogenetic characters with character states transforming according to linearly ordered and reversible pathways. Trees of proteomes can be generated from the matrices of phylogenetic characters. They are not used in this paper but are largely congruent with traditional classification. B. Evolution of tRNA structure and function. The ancient ‘top half’ of tRNA embeds a ‘operational code’ in the identity elements of the acceptor arm that interact with the catalytic domain of aaRSs through class I and II modes of tRNA recognition. The evolutionarily recent ‘bottom half’ of tRNA holds the standard code in identity elements of the anticodon loop that interact with anticodon-binding domains of aaRSs. The flow diagram below describes the phylogenetic reconstruction of trees of tRNA substructures (ToSs). The structures of rRNA molecules were first decomposed into substructures, molecules. Structural features (e.g., length, Shannon entropic descriptors) of substructures such as helical stem tracts and unpaired regions are coded as phylogenetic characters and assigned character states according to an evolutionary model that polarizes character transformation towards an increase in conformational order (character argumentation). Coded characters (s) are arranged in data matrices, which can be transposed for further cladistic analyses (e.g., to produce trees of substructures). Phylogenetic analysis using maximum parsimony optimality criteria generates rooted phylogenetic trees of tRNA molecules. Embedded in trees of domains and trees of tRNAs are timelines that assign age to molecular structures and associated functions. C. Culling of PDB sequences for calculation of amino acid frequencies and dipeptide counts. Dipeptides define concatenated 2-mer amino acid sequences.

The evolutionary accretion of structure is the process by which molecules add parts such as substructures or structural modules (domains) to their molecular makeup. The process can take millions of years and is usually driven by improvements in biological function and stability [5.6]. Timelines that describe the gradual appearance of domain structures in evolution have shown that the editing and anticodon-binding domains of aaRSs are late additions to the central catalytic role of their aminoacylation domains [6], [7]. In these timelines, the relative ages of domains at three different levels of the structural hierarchy defined by the structural classification of proteins [8], fold (F), fold superfamily (FSF), and fold family (FF), are derived from phylogenomic trees reconstructed from a census of domain structure in hundreds of fully sequenced genomes (reviewed in [5]). The phylogenomic strategy, which is summarized in Figure 1A, reconstructs history with high predictive power. The ages of protein domains explain known patterns of structural change [9] and organismal diversification [10], early evolution of molecular functions [11], links between geochemistry and metallome evolution [12], and the definition of a molecular clock that matches fossil and geochemical records [13], [14]. For example, the age of single-domain enzymes established that aerobic metabolism appeared ∼2.9 billion years (Gy) ago [13] and that Mn catalase was the most likely culprit of planet oxygenation [14], findings that align with inferences from geology and geochemistry [15]. Domain history is also compatible with studies of physical clustering of genes in genomes [16], links between ancient sequence motifs in loops and domain structures [17], conservation of loop motifs [18] and an analysis of gene birth, transfer, duplication and loss in gene families across evolutionary history [19]. Network connectivity in these studies suggest that a central metabolic core that includes nucleotide/phosphate binding functions typical of ABC transporters and nucleotide interconversion reactions, is more ancient that rings of gene neighbors and clusters of ancient motifs organized around aaRSs and even more ancient than a gene expansion enriched in electron-transport and respiratory functions that occurred ∼2.9–3.3 Gy ago. The emergence of the most ancient functions in timelines of domains suggests that the first proteins were most likely hydrolase and transferase enzymes involved in nucleotide interconversion, storage and recycling of chemical energy through high energy phosphate transfer [20]. These enzymes harbor the P-loop containing nucleoside triphosphate (NTP) hydrolase fold (c.37), specifically the ABC transporter ATPase domain-like and the extended and tandem AAA-ATPase domain FFs [7], confirming previous inferences at other levels of the structural hierarchy [5], [9], [20]. Remarkably, the evolutionary age of domains correlates with the age of associated molecular functions inferred directly from hundreds of thousands of terminal ontological terms of molecular functions and biological processes [11]. These studies suggest again that metabolism preceded translation and showed that the oldest proteins had ATPase, GTPase, and helicase activities. We note that the primordial ATPase FFs have the potential to use the energy of nucleotide binding and hydrolysis for mechanical work, which is generally used to move polypeptides and nucleotides [21]. The fact that enzymatic catalysis precedes aminoacylation, and aminoacylation precedes RNA recognition congruently in retrodictive and nomothetic analyses of structural, functional and genomic data is remarkable and has implications for this study – congruence should be considered the most powerful statement of evolutionary biology.

The phylogenomic strategy can also link evolution of proteins and RNA (Figure 1). The age of ribosomal protein domains was found to coevolve with the age of interacting rRNA substructures, revealing a complex interplay of recruitment and accretion patterns, and unexpectedly, the relatively late molecular origins of the ribosome [22], [23]. In tRNA, the ‘bottom half’ of the molecule embeds the ‘classical’ (standard) code in the anticodon arm and its identity elements in the anticodon loop interact with anticodon-binding domains. This bottom half is distal (∼76 Å) to the ‘top half’ that harbors the ‘operational’ code in the acceptor stem [24], the discriminator N73 base and other important identity elements needed for amino acid editing and aminoacylation [25]–[27](Figure 1B). Phylogenetic studies of the sequence and structure of thousands of tRNA molecules showed that the bottom half is also distal in time (0.3–0.4 Gy) to the more ancient top half, which grew by slow substructural accretion [28]. The age of cognate tRNA also showed that amino acid charging and encoding had separate histories and involved episodes of structural recruitment [28], [29]. These studies revealed that the acceptor arm tRNA structures charging Tyr, Ser and Leu evolved earlier than anticodon loop structures responsible for amino acid encoding [29]. Thus, the very first amino acid specificities of archaic aaRSs must be imprinted in the ancient catalytic and editing domains that interact exclusively with the top half and the variable loop and elbow region of tRNA.

Here we focus on the accretion of proofreading and specificity domains of aaRSs and their interaction with evolving tRNA. We find that the most ancient aaRS domains harbor archaic dipeptidase and ligase activities, suggesting that the primordial peptides and polypeptides they originally produced conditioned the amino acid makeup of modern proteins. To test this hypothesis, we study the sequential overlap of dimers in the amino acid sequence of proteins and consider that these ‘dipeptide’ constituents are relics of ancient amino acid composition. Each dipeptide defines one of ∼400 pairs of two sequential residues or ‘peptide bond types’ that make up protein sequence. We show that dipeptides of protein domains appearing in evolution before the first anticodon-binding domain are significantly enriched in amino acids that are subject to aaRS editing. Remarkably, results drawn directly from phylogenomic and structural information uncover a hidden link between the emergence and expansion of the genetic code and protein flexibility. This link provides important clues to understand one of the most challenging problems that exist in biology.

## Results

### Research Goals and Strategy

We use timelines that describe the gradual appearance of protein domains in the protein world and substructures in RNA molecules to: (i) unfold the history of aaRS domains and their associated functions, (ii) determine if aaRS domains and tRNA molecules coevolve, (iii) retrodict the origin and evolution of the genetic code, and (iv) find connections between the rise of genetics and patterns embedded in the structure of proteins. Our approach is mostly ideographic (historical, retrodictive) and is strongly grounded in cladistics and reciprocal Hennigian illumination [30]. Cladistic theories are compound and multidimensional hypotheses of the relationship (phylogenies) of entities (taxa) and the evolutionary transformation of the biological attributes (phylogenetic characters) that define those relationships (models of evolution). While phylogenies make assertions about singular events in history (retrodictions), the principle of reciprocal illumination evaluates how each primary homology statement that describes the evolution of attributes [31] agrees with the overall favored evolutionary hypothesis obtained from all available data (e.g. gene content in genomes and the tree of life [32]). Agreements are used to reformulate homology hypotheses in an iterative framework of maximization of explanatory power [33] that links phylogenetic analysis and the Popperian pillars of content of theories and degree of corroboration [34]. In this framework, increasing explanatory power over background knowledge is obtained through test and corroboration and not through verificationist strategies that seek to increase support of nodes in trees. In our case, protein domain definitions, RNA substructures and dipeptide signatures are used as ‘basic’ evidential statements within the Popperian framework. They should be regarded as agreed upon conjectures of perceived similarities that are accepted as fact for the duration of the study, are strengthen by reciprocal illumination, and help define useful homologies, trees and timelines.

### Protein Domain Accretion in aaRSs

**Figure 2:**
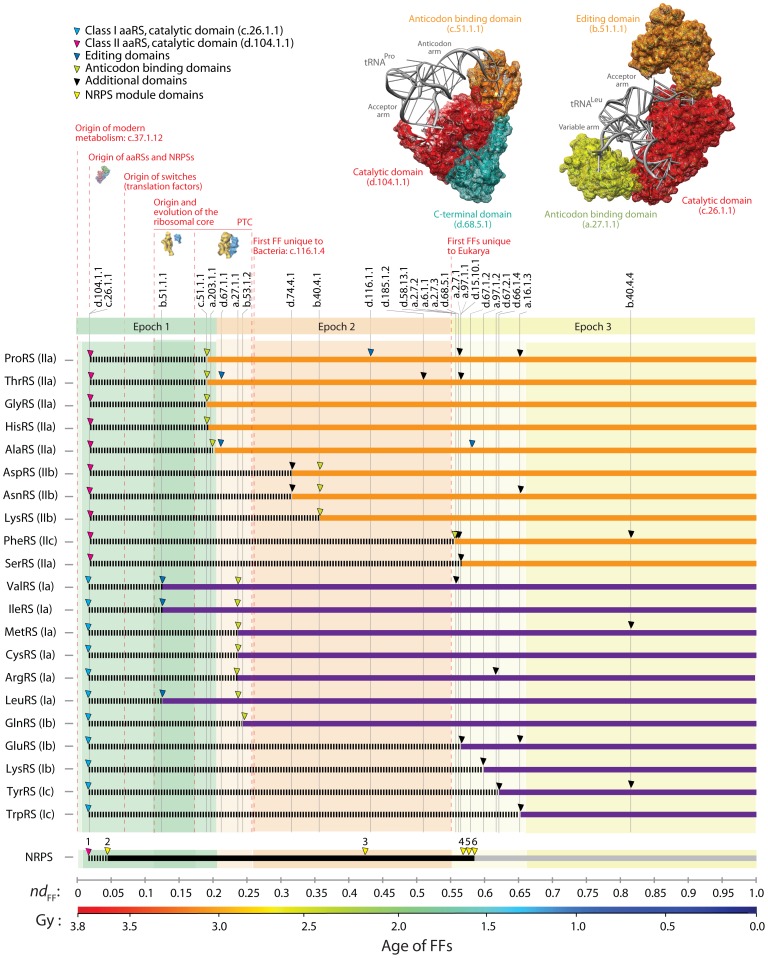
Evolutionary accretion of domains in aaRS enzymes. The age of domains for aaRS enzymes (arrowheads) were mapped along a timeline of domain appearance generated from a phylogenomic analysis of FF structures in 420 free-living organisms (Figure S1). The three epochs of the protein world are shaded and the evolution of NRPS modules is given as reference together with other relevant landmarks. Domains were identified with *concise classification strings* [8]. A molecular clock of domain structures places the relative timeline in a geological time scale [13]. The inset shows examples of evolutionary accretion of aaRS domains. Structural models of ProRS from *Thermus thermophilus* complexed with tRNA^Pro^ (PDB entry 1H4S) and LeuRS from *Pyrococcus horikoshii* complexed with tRNA^Leu^ (1WZ2) with catalytic, editing, anticodon-binding and accessory domains colored according to their age of origin.

The relative age of individual domains (*nd*_FF_) was calculated from a published tree of domain structures [6], [7] as the number of nodes from a hypothetical ancestral FF structure at the base of the rooted tree, given in a relative 0–1 scale. The tree (reconstructed using the strategy of Figure 1A) describes the evolution of 2,397 FFs and was obtained from a phylogenomic analysis of 754,867 inferred structures (Figure 2). *nd*_FF_ was used to construct a timeline of domains, with time flowing from the origin of proteins (*nd*_FF_ = 0) to the present (*nd*_FF_ = 1). These *nd*_FF_ values are good proxy for geological time when trees of domains are used as molecular clocks [13]. We mapped the age of 27 aaRS FFs, for each aaRS (Figure 2) and globally (Figure S1). We find that catalytic aminoacylation domains are among the eight most ancient FFs (*nd*_FF_ = 0–0.024), all of which harbor Rossmann-like α/β/α-layered topologies [7]. The FF of class II enzymes (c.26.1.1) appeared concurrently with the GTP-binding domain of elongation and initiation factors, the G protein domain (c.37.1.8), ∼3.7 Gy ago at *nd*_FF_ = 0.020. The FF of class I enzymes (d.104.1.1) appeared immediately after at *nd*_FF_ = 0.024. Our timelines indicate that the process of domain accretion started with the CP1 ‘ValRS/IleRS/LeuRS editing domain’ FF (b.51.1.1; *nd*_FF_ = 0.126) ∼3.3 Gy ago and that class II enzymes were the first to add anticodon-binding domains ∼ 3 Gy ago (c.51.1.1; *nd*_FF_ ∼ 0.196; a.203.1.1; *nd*_FF_ ∼ 0.200) (Figure 2). Domains of class I enzymes closely followed (a.27.1.1; *nd*_FF_ ∼ 0.241; b.53.1.2; *nd*_FF_ ∼ 0.249). Accretion spanned all three major epochs of the protein world [10] and over 2.6 Gy of evolution (Figure 2; Figure S1).

### Coevolution of tRNA and aaRS Domains

**Figure 3:**
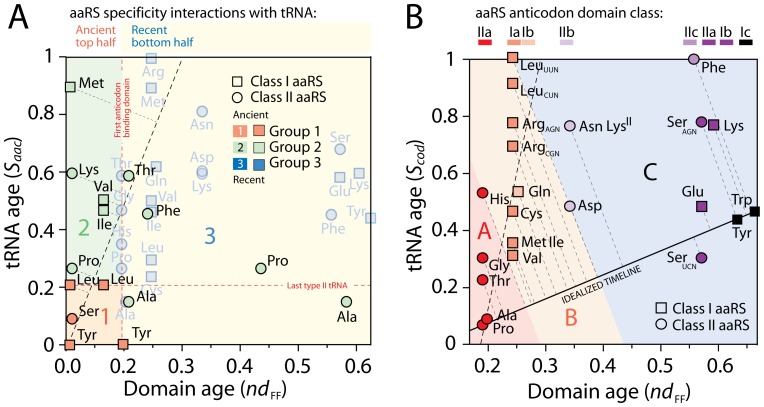
Coevolution of aaRS domains and cognate tRNA. A. The ages of catalytic and editing domain FFs (*nd*_FF_) interacting with type II tRNA (*Group 1*) and type I tRNA (*Group 2*) and the age of anticodon-binding domain FFs (*Group 3*; provided only as reference) were plotted against the age of tRNA isoacceptors (*S*_aac_). The significant correlation (*P*<0.0067) unfolds an evolutionary timeline of early domain function. The time period spanning the most ancient functions (class I TyrRS) and the most recent trans-editing function (Ala-X of AlaRS) involves ∼2 Gy of evolution. B. Coevolution of anticodon-specific tRNAs (*S*_cod_) and anticodon-binding domain FFs (*nd*_FF_) is significant for the early start of the code (dashed line; *F* = 20.8; *P*<0.001), but are followed by episodes of tRNA structural recruitment. An ‘idealized’ timeline partitions code expansions in three age groups (A, B and C).

Only 10 aaRSs harbor editing functions, and most amino acid sieving functions of editing domains appeared before anticodon-binding domain recognition of identity elements in the anticodon arm of tRNA (Figure S1). We explored coevolutionary patterns of (i) amino acid charging involving primordial aaRS domains with editing functions and tRNA isoacceptors, and (ii) genetic encoding involving more recent aaRS anticodon-binding domains and anticodon-specific tRNA (Figure 3). We note that the average Kyte-Doolittle hydropathy index of amino acids involved in the first group (1.2) exceeded by far that of amino acids of the second group (−0.22), highlighting their hydrophobic nature and a possible association of the encoded amino acids to primordial membranes. In these studies, the age of isoacceptor and anticodon-specific tRNA was obtained by phylogenetic constraint analyses [29] as described in Figure 1B. Figure 3A plots the age of tRNA isoacceptors (*S*_aac_) against the age of aaRS domains (*nd*_FF_) endowed with editing activities. We assume the age of the FF domain is the age of the associated molecular function [22], [23]. Light-shaded data points for anticodon-binding domains are late evolutionary developments and are only provided as reference. The plot reveals co-evolution of tRNA and aaRS domain structure (lineal regression: editing set, excluding trans-editing recruitments; *S*_acc_ = (2.40±0.79) *nd*_FF_; *R^2^* = 0.741; *F* = 9.10; *P*<0.0117; total set; *S*_acc_ = (1.17±0.17) *nd*_FF_; *R^2^* = 0.814; *F* = 47.87; *P*<0.0001). The regression line unfolds an evolutionary timeline of archaic editing functions. TyrRS, SerRS, and LeuRS interact with the oldest cognate tRNAs, type II tRNA molecules harboring a long variable loop necessary for tRNA recognition. Their catalytic domains appear very early at *nd*_FF_ = 0.020–0.024. The most ancient editing functions are pre-transfer and post-transfer mechanisms mediated by TyrRS, SerRS, LeuRS, ProRS, LysRS and MetRS (Figure S1). *S*_aac_ values indicate that IleRS and ValRS CP1 domains were recruited from the LeuRS variant. The D-loop of tRNA^Ile^ does not make direct contact with the CP1 domain but impacts tRNA conformation and is critical for the editing activity of IleRS, not for aminoacylation [35]. Its requirement at this very early evolutionary stage suggests that a cloverleaf tRNA structure was already operational at *nd*_FF_ >0.126. Figure 3B plots the age of anticodon-specific tRNAs (*S*_cod_) as a function of the age of anticodon-binding domain FFs (*nd*_FF_) and reveals coevolution during the *nd*_FF_ = 0.196–0.249 interval and late tRNA structural recruitments occurring initially in parallel with recruitment of ribosomal protein domains in the ribosome. An ‘idealized’ timeline partitions the code in three anticodon tRNA-aaRS binding interaction expansions: (A) Pro–Ala, Thr, Gly and His, from ancient to derived (involving class IIa anticodon-binding domains); (B) Val, Met–Ile, Cys, Gln, Arg and Leu (involving class Ia and Ib anticodon-binding domains); and (C) Asp, Asn–Lys (class II), Ser, Glu, Tyr, Lys (class I) and Phe–Trp, resulting from late recruitments.

### Mapping Domain Groups onto Genetic Code Representations

**Figure 4:**
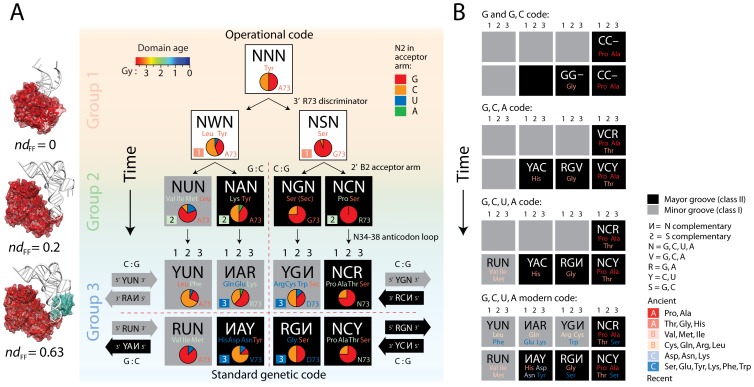
Origin and evolution of the genetic code. A. Inception of the ‘operational’ code. Mapping of amino acid charging functions onto a binary decision-tree and a condensed *vis-á-vis* complementarity representation of the genetic code. Cells are indexed with *Group 1, 2 and 3* domain inception, discriminator base identity, and nucleotide composition (pie charts) of the N2 position of the acceptor stem of tRNA. In the right, structural models of TyrRS (1H3R) interacting with tRNA^Tyr^ and an acceptor-minihelix illustrate a possible evolutionary route of domain growth and accretion as the binary tree unfolds in evolution (domains are colored with corresponding geological age). B. Evolution of the ‘standard’ code. Ancestries define a timeline of early genetic code expansion in the condensed *vis-á-vis* code representation with major and minor groove modes of tRNA recognition. The mappings take into consideration the alphabet and number of anticodon positions that are most parsimonious and anticodon loop identity elements. Note that Pro, the founder, already uses 2nd and 1st code positions (identity elements G35 and G36) and that the first use of 3rd codon position (G34) occurs first with Thr and then His (the last two initial recruitments of c.51.1.1) when the alphabet expands to the triplex code. Also, the Yin-Yang complementarity pattern is fulfilled with the last recruitment of a.27.1.1 once the modern tetraplex code is in place.

The coevolutionary timelines allowed binning aaRS domains conservatively into three groups, ancient domains with editing functions interacting with the basal type II tRNA charging Tyr, Ser and Leu (*Group 1*, the most ancient) and type I tRNA (*Group 2*, of intermediate age), and more modern domains interacting with the bottom half of tRNA (*Group 3*; recent anticodon-binding domains) (Figure 3). We mapped the age of these three domain groups onto Delarue’s modified binary decision-tree representation of the genetic code [36] coupled to a condensed code representation [37] that places complementary codons *vis-á-vis* each other and describes major and minor groove modes of tRNA recognition by aaRSs (Figure 4A; extended version can be found in Table S1). Each step of the binary decision process reduces ambiguity, is most parsimonious in changes of the N73 discriminator of the acceptor stem, and follows the arrow of time defined by the coevolution plot and the onset of the three domain groups. According to the timeline, the operational code [24] starts with aaRSs that interact with primordial tRNA hairpin loop structures, are homologous to TyrRS catalytic domains, and have emerging A73 discriminator identity functions. A simple A to G mutational change in the alphabet of the N73 discriminator splits the emerging code in two lineages in the tree (complementary to future NWN and NSN codons) with potential to preferentially charge Leu and Ser. These lineages are future founders of groove modes of tRNA recognition and the antisense complementarity of the code. Charging of Ser involves for the first time a G2:C72 base pair in the acceptor arm that is known to be a weak identity element important for aminoacylation and separates Ser from Tyr and Leu specificities [25]–[27]. The next step of the decision tree is driven by bias in the makeup of position 2 of tRNA that defines the operational code [38] and by other emerging identity elements of the acceptor arm important for editing and aminoacylation. As previously reported [38], we find that pairs of complementary codons in the *vis-á-vis* representation exhibit a coordinated complementarity of C2-G72 and G2-C72 base pairs in the acceptor stem (with an exception of only 3 of 32 comparisons) (Table S1). Within these complementary base pairings, NUN-NAN and NGN-NCN codons already show distinct overrepresentation of G or C in position 2 of the acceptor stem. This step of the evolutionary decision tree is the first of a number of ambiguity reduction steps associated with identity elements in a primordial anticodon loop that probably appeared at this stage of tRNA-aaRS coevolution (see below). Finally, at the base of the decision tree (now shown in the *vis-á-vis* code representation), complementary base pairings are overrepresented in G for major-major groove RGN-NCY and major-minor YGN-NCR comparisons, while being drastically underrepresented in the major-minor RUN-NAY and slightly underrepresented in the minor-minor YUN-NAR complementarity relationships. Representation patterns in the GC alphabet of the operational code are mostly due to aaRSs with amino acid editing properties that are ancient, suggesting an archaic imprint of tRNA identity elements in emerging translation (Table S1). Since an analysis of minimizing risks of confusion in the recognition of complementary anticodon-codon pairs [37] revealed a possible evolutionary progression in the establishment of the genetic code that follows the RGN-NCY, YGN-NCR, RUN-NAY and YUN-NAR pairs, in that order (Table S2), our patterns of enrichment suggests this evolutionary progression is remarkably recapitulated by the gradual establishment of the GC alphabet of the operational code.

To unfold the origin and evolution of the ‘standard’ genetic code at the base of the decision tree, we mapped the ancestries of anticodon tRNA-aaRS binding expansion groups A, B and C onto the condensed *vis-á-vis* code representation [37](Figure 4B), a degenerate genetic code table (Figure S2), and Venn diagrams of amino acid properties (Figure S3). Phylogenomic mappings showed the centrality of the second and first codon positions [29], early major groove recognition [37], and a progression that starts with a code based on C that later expands by adding G, A, and U to its makeup, in that order. Early codes were associated with small and hydrophobic amino acids (Figure S3). We note the enrichment of the operational code in G during its early inception (Figure 4A), which is complementary to the inferred most ancient word of the vocabulary. However, the origin of the standard code probably involved both G and C and the first and second codon bases responsible for primordial coding of Pro and Ala (Figure 4B).

### Evolution of Identity Element Specificity in tRNA

An analysis of identity elements in cognate tRNA and their respective role in aminoacylation [25]–[27], estimated by loss of aminoacylation efficiency upon mutation, showed that type II tRNA elements interacting with *Group 1* domains are substantially less affected by mutational manipulation than type I tRNA elements interacting with *Group 2* and *3* domains (Figure S4). Moreover, the ratio of acceptor-to-anticodon arm identity elements interacting with *Group 1* domains is also substantially larger. As expected, the oldest group of aaRSs (*Group 1*) has retained ancestral recognition features of limited specificity, which in biochemistry are considered ancestral [39], [40]. These features belong to the acceptor stem, especially the N73 discriminator base and the N4:N69 base pair that is uniquely recognized by SerRS, LeuRS, IleRS ValRS and MetRS (Figure S4).

### Genetic Code Expansion Unfolds Constraints Imposed by the Secondary Structure of Proteins

We mapped code expansion in sense-antisense codon exchange graphs that retain secondary structure information [41]. An analysis of secondary structure in 6,828 structural models shows code expansion first occurred through tRNA major groove recognition in complementarity pairs implementing amino acids important for turn motifs, then involved major/minor groove recognition important for β-strand formation, and finally minor groove recognition important for α-helical local structure of proteins (Figure S5A). Patterns also show the initial importance of helical (and turn) structure in early protein evolution (Figure S5B).

### The Amino Acid Composition of Proteins Supports a Direct Link of Archaic Peptide Biosynthesis and Code Specificities

Structures homologous to the most ancient *Group 1* catalytic domains have atypical functions. Amino acid-[Acyl-Carrier-Protein]-ligases (aaACPLs) homologous to SerRSs aminoacylate 4′-phosphopantheteinyl and other prostetic groups of carrier proteins [42], a function that is typical of non-ribosomal protein synthetase (NRPS) modules. Cyclodipeptide synthases (CDPSs) homologous to TyrRSs make cyclodipeptides from charged tRNAs specified by *Groups 1 and 2* domains (e.g., [43]). Structural alignments of aaACPLs and CDPSs to close aaRS structural neighbors show that the structural cores and crucial sequence sites that contact ligands are highly conserved (Figure S6) and suggest aaACPL and CDPS structures are relics and founders of archaic biosynthetic activities. Since dipeptide synthases require charged tRNA to function, we conjecture that archaic aaRSs capable of aminoacylation and peptide bond formation acted in concert to perform primordial protein biosynthesis before the existence of the ribosome [7].

**Figure 5:**
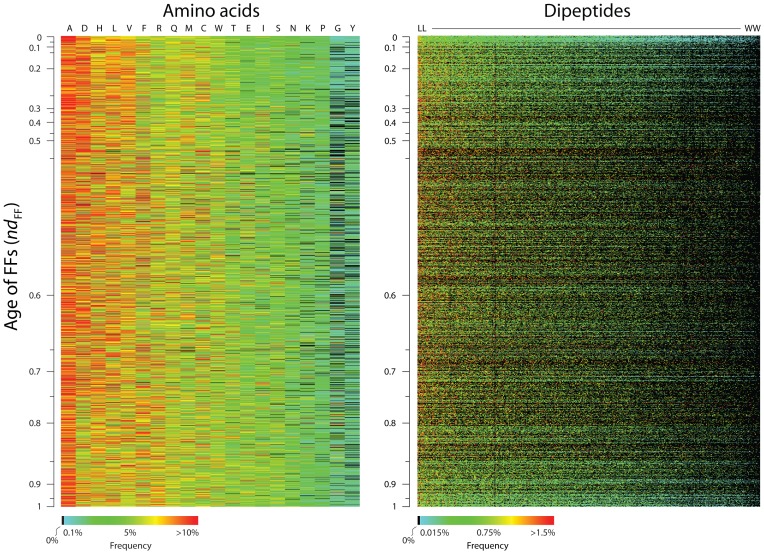
Evolutionary heat maps describing the amino acid and dipeptide compositions of FF domain structures of different age. A. Frequency of amino acids in FFs. The color array of 29,480 cells (1,475 rows×20 columns) describes the amino acid composition of 1,475 FFs along the evolutionary timeline. Columns represent the 20 standard amino acids ordered (from left to right) according to average amino acid frequency and rows represent FFs ordered (from top to bottom) according to domain age (*nd*_FF_ = 0 ∼ 1). B. Frequency of dipeptides in FFs. The color array of 589,600 cells (1,475 rows×400 columns) describes the 400-dipeptide composition of FFs along the timeline. Columns represent dipeptide types ordered (from left to right) according to average frequency (from LL to WW) and rows represent FFs ordered according to age. The heat maps confirm the existence of non-random patterns of amino acid and dipeptide compositions along the evolutionary timeline of FFs and reveal unique signatures of amino acid and dipeptide use in FFs. Amino acids are described with single-letter codes.

In order to support this claim we focused on the expected impact of dipeptide synthesis on the peptide bond (dipeptide) makeup of protein structures (Figure 5). Dipeptides provide unique signatures of fold structure [44] with predictive power matching that of domains [45]. However, to avoid modern evolutionary effects of domain recruitment and rearrangements and discount possible structural ambiguities, we selected 2,384 single domain proteins with 1,475 FF assignments from our initial set of 204,531 structural entries (Figure 1C). This allowed accurate tracing of dipeptides in high-quality structural models. Simple heat map representations show amino acid monomer and dipeptide composition for every FF mapped along the timeline (Figure 5). The 20-dimensional amino acid and 400-dimensional dipeptide ‘signature’ vectors of FFs in these evolutionary heat maps uncover compositional signatures that act as diagnostic ‘fingerprints’ of individual domain structures. Mining of signature patterns reveal strong and general biases in the use of amino acids and dipeptide types. Unique patterns of amino acid and dipeptide use in FFs have considerable potential for fold recognition in structural bioinformatics applications (Wang et al., ms. in preparation). Heat maps show a bimodal distribution of patterns of monomer and dipeptide use; the most ancient and most recent FFs exhibit a general tendency towards both lowest abundance and maximum diversity. These tendencies were maximal at *nd*_FF_ <0.1 and *nd*_FF_ >0.95. The most abundant amino acids throughout the timeline was Ala, followed by Asp, His, Leu and Val, which were less affected by the bimodal pattern. Similarly, the most abundant dipeptide was Leu-Leu, followed by Ala-Leu, Ala-Ala, Leu-Ala, Glu-Leu, Leu-Glu, Leu-Lys, Glu-Glu, Val-Leu, Ala-Glu, Gly-Ser, Leu-Gly, Gly-Leu, and Glu-Ala, in that order, all of which were represented at frequencies that were 25% higher than all other dipeptides. Gly and Tyr were absent in many FFs, and absences of many dipeptides were pervasive and spread throughout the timeline in a bimodal pattern. The average Kyte-Doolittle hydropathy index of the most frequent half of amino acids was −0.27, while that of the less frequent was −1.21. Since the average amino acid index is −0.49, the large values of frequent amino acids indicate their high relative hydrophobicity. Similarly, the average index of the amino acid components of the 14 most frequent dipeptides listed above was 0.93, again stressing the importance of the hydrophobic makeup of these residues.

**Figure 6:**
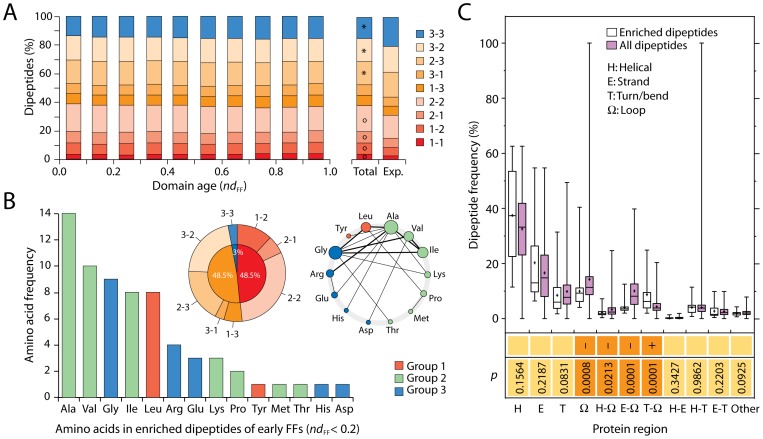
Dipeptide makeup of ancient proteins. A. The distribution of dipeptide compositions in proteins shows remarkable conservation along the FF timeline. Stacked column charts describe the 408 possible dipeptides (combinations of two amino acids) corresponding to 9 sets specified by *Groups 1, 2* and *3* aaRS structures (1-1, 1-2, 2-1, etc). The stacked columns on the right display the general distribution pattern of dipeptides in the dipeptide sets for all 2,384 sequences and the expectation of dipeptide set distributions calculated by free permutation. Circles and asterisks represent groups that are over- or underrepresented, respectively, following *χ*−square statistical contrasts. B. Ancient FFs appearing before anticodon-binding domains (*nd*_FF_ ≤0.2) were significantly enriched (*P*<0.01) in dipeptides composed of amino acids specified by the ancient editing domains (*Group 1* and *2*). The bar plot shows the amino acid frequencies of the 33 enriched dipeptides, the doughnut chart describes enriched dipeptide set compositions, and the network displays dipeptide makeup, with peptide bonds (edges, weighed by number of dipeptide types) connecting participating amino acids (nodes, with size proportional to connections). C. Mapping of enriched dipeptides in protein structures. Box-and-whisker plots describe the distribution of the 33 dipeptides that are significantly enriched in early FFs (*nd*_FF_ ≤0.2) versus that of all dipeptides in regular and non-regular structural regions of the 2,384 protein sequences analyzed. Regular structures include helical regions (H) with α-helix (h), 3_10_-helix (g) and π-helix (i) elements, strand regions (E) with β-strand (e) and β-bridge (b) elements, and turn/bend regions (T) with turns (t) and bends (b). Non-regular (unstructured) regions include loops (Ω). PBT amino acids can span different regions. Statistical differences between PBT were defined by p-values of Mann-Whitney non-parametric tests. Increases and decreases in central tendencies for the ancestral proteins are indicated with+and – signs, respectively, for structural sets with significant associations.

Since amino acid and dipeptide signatures manifest in individual folds along the timeline, any composition bias must critically influence emerging protein structure. The analysis revealed a statistically significant progression of frequencies of amino acids specified by *Group 1*, *2* and *3* structures, constant along the FF timeline (ANOVA; *p*<0.0001), with *Group 1* amino acids showing the largest and *Groups 3* the smallest frequencies (Figure S7). Since larger frequencies of molecular features that are globally reused are generally considered ancestral [46], this result provides additional independent support to the ancient origin of amino acids specified by editing *Group 1* and *2* domains. An analysis of dipeptide counts in this protein set showed the relative proportion of amino acids making peptide bonds (Figure 6A). Again, dipeptides involving amino acids specified by editing *Group 1* and *2* domains were overrepresented and those that involved *Group 3* domains were underrepresented over statistical expectations (χ−square test: *p*<0.0001; df, 34,480, 8), with patterns that were remarkably consistent throughout the timeline. Thus, dipeptides display ancestral specificities of aaRS domain history.

**Figure 7:**
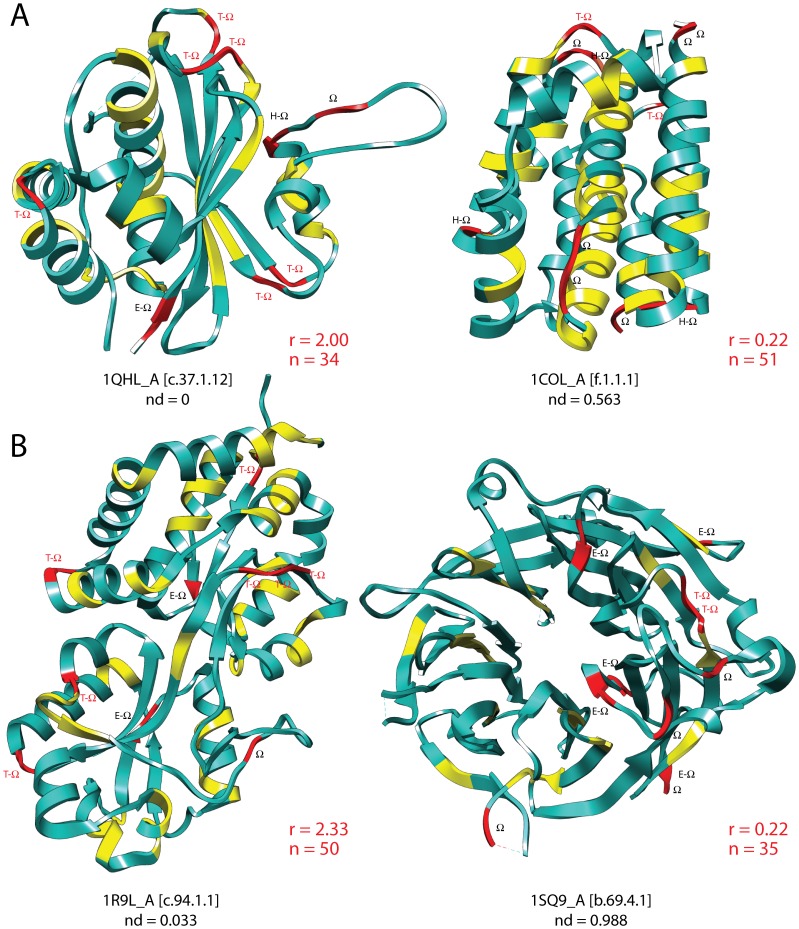
Mapping enriched dipeptides in the structural model of proteins of ancient and recent origin. A. The N-terminal domain of *Escherichia coli* MukB chromosome partitioning protein (1QHL), which harbors the most ancient FF structure (c.37.1.12), is compared to the pore-forming domain of colicin A (1COL), which harbors the more recent colicin FF structure (f.1.1.1). B. The glycine betaine-binding periplasmic protein ProX (1R9L), which harbors the ancient phosphate-binding protein-like FF (c.94.1.1), is compared to the antiviral protein Ski8 (1SQ9), which harbors the very recent WD40-repeat FF (b.69.4.1). Dipeptides significantly enriched (*P*<0.01) in ancient proteins are labeled in yellow and red, with red regions involving loop segments. Ancient proteins are on average enriched in dipeptides located in regular structures (segments in yellow) and are depleted in dipeptides located in loops (Ω)(segments in red), except for those in boundaries with turns (T- Ω) (with labels in red). A ratio of the number of T- Ω dipeptides to other dipeptides in loops (*r*) clearly shows the rigidity of loops of ancient proteins. With exceptions (e.g. the comparison of MukB and colicin A), the total number of enriched dipeptides (n) reveals impoverishment of enriched dipeptides in evolution. The structures that are shown are representative pairs of typical structures of similar total length belonging to the ancient and derived groups. Except for MukB, they were selected at random. We note that protein class does not affect the dipeptide distribution trends we report.

To confirm this important finding, we compared dipeptides appearing before anticodon-binding domains (*nd*_FF_ ≤0.2) to those in the entire timeline and identified dipeptides that were specifically overrepresented using the hypergeometric distribution (Table S3). A total of 33 dipeptides were significantly enriched in ancient proteins (*P*<0.01). With only one exception, half of enriched dipeptides involved one and the other half involved two amino acids specified by the ancient editing domains (Figure 6B). Remarkably, there were no enriched dipeptides involving two *Group 1* amino acids, and Ser was entirely absent, suggesting archaic SerRSs did not partake in very early tRNA aminoacylation, acting perhaps as aaACPL-like ligases instead. Only 14 amino acids were part of enriched dipeptides, 9 of which were *Group 1* and *2* amino acids and constituted 73% of dipeptide makeup. Three of the other 6 amino acids (Gly, Glu and Asp), together with Ala, Val, Ile, Leu, were highly represented in the Urey-Miller experiments [47] and are considered prebiotically abundant and prone to prebiotic dipeptide formation [48], [49]. The average Kyte-Doolittle hydropathy index of the amino acids in the overrepresented set was 1.2, paraphrasing the index of amino acids subject to editing (see above). Graphs of dipeptide makeup showed Ala and Gly, and then Val, Ile and Leu, were hub-like (Figure 6B). Mappings of ancient enriched dipeptide onto secondary structures revealed they were not significantly biased (non-parametric Mann-Whitney test; p>0.05) towards regular structural regions such as helical, extended, or turn regions (Figure 6C). However, there were significant biases (p<0.05) against loop regions and toward loop-turn boundaries. Figure 7 shows dipeptides mapped onto representative structures.

## Discussion

### An Early Operational Code Gives Way through Coevolution to Modern Genetics

Translation specificity is the ultimate culprit of the genetic code and the ‘memory’ needed for modern genetics. Without it proteomes become statistical (e.g., [50]). In vitro studies have shown that discrimination against non-cognate substrates is maximal in aminoacyl-tRNA synthesis (1/200–10,000 misincorporated amino acids and 1/>10,000 misincorporated tRNA), unknown but probably significant for EF binding, and minimal for aaRS editing, aaRS resampling, and ribosomal tRNA recognition and proofreading (1/∼10–100 for tRNA substrates) [3]. Genetic code safekeeping has been for the most part entrusted to aaRSs. Since engineered minimal cores of catalytic domains of aaRSs exhibit unusually strong enzymatic activities with ∼9 out of ∼14 orders of magnitude in rate acceleration [51], [52], these highly conserved doppelgängers (urzymes) hold the key to the origin of translation. In this study, evolutionary timelines reveal that aaRS domains and tRNA structures coevolve and are later recruited into various functions. Coevolutionary patterns show that aaRSs originated in catalytic domains, which make up urzymes and harbor biosynthetic functions, and that these archaic structures slowly gained specificity by building interactions with primordial tRNAs. This challenges the long-standing notion that code inception preceded protein biosynthesis.

We find that tRNA isoacceptors coevolve with aaRS domains with pre-transfer and post-transfer editing and trans-editing activities (Figure 3A), which were the first to appear ∼3.7 Gy ago. The most ancient of these editing structures, present in the catalytic domains of *Group 1* TyrRS, SerRS and LeuRS (*nd*_FF_ <0.196), involved the oldest type II cognate tRNAs, which harbor a long variable loop necessary for tRNA recognition. An analysis of identity elements in the acceptor stem of tRNA suggests that these archaic charging specificities are founders of mirror modes of tRNA acceptor stem recognition and the ancient operational code (Figure 4A). Class I and II aaRS enzymes appear almost concurrently in our timelines, fulfilling assumptions of three evolutionary models of aminoacylation stereochemistry (Figure S8). Class I enzymes approach tRNA from its minor groove and aminoacylate the 2′-hydroxyl group of the terminal adenosine of the tRNA acceptor arm (except TyrRS), while class II enzymes (except PheRS) do so from the major groove esterifying the corresponding 3′-hydroxyl group [53]. Our timelines indicate that this fundamental stereochemical behavior, determined by the crucial ‘discriminator’ N73 base of tRNA [54], arose when archaic TyrRS-like catalytic structures with major groove recognition abilities developed class I minor grove recognition capabilities in primordial LeuRS enzyme derivatives (perhaps via head-to-tail amino acid complementarity), setting up in motion the ‘yin-yang’ complementarity pattern [37] of the code (Figure 4A) and patterns of secondary structure information embedded in codon exchange graphs [36](Figure S5A). Primordial coding for turns linked to major groove recognition gave way to coding for helical segments preferred by minor groove recognition (Figure S5A). We note that tRNA^Ser(CGA)^ in selected fungal species can be aminoacylated with Ser and Leu by SerRS and LeuRS [55]. These tRNAs with multiple aminoacylation identities are probably vestiges of pervasive crosstalk in ancient aminoacylating enzymes and the result of the weakness of the primordial identity elements (Figure S1) responsible for the first split in the code’s decision tree (Figure 4A). We also find that the GC alphabet (Table S1) of the ancient operational code [38], embedded in the codon/anticodon-like first three base pairs of the acceptor stem, conditions the evolutionary appearance of ancient *Group 2* amino acid charging specificities, which involve Val, Ile Met, Lys, and Pro.

Interactions of tRNA with the CP1 domain [35] suggest the D arm was already present 3.3 Gy ago, which is derived compared to the acceptor stem [28]. The late appearance of domain-encoded anticodon specificities in well over half of aaRSs (*nd*_FF_ ∼ 0.2–0.25) confirms that the full bottom half of tRNA and its anticodon loop identity elements, especially complementary triplets (anticodons), unfolded completely ∼3 Gy ago (Figure 1B). Protein-RNA coevolutionary interactions probably fixed specificities that were originally established by the ancient operational code, enabling genetic code expansions by structural recruitments and mutational change (Figure 3B). This late development coincided with the origin of ribosomal proteins at *nd*_FF_ = 0.114 and the inception of the ribosomal peptidyl transferase center (PTC) responsible for modern protein synthesis and the establishment of a fully functional ribosomal machine ∼2.8–3.1 Gy ago [23], together with pathways of amino acid [14] and purine nucleotide biosynthesis [56]. This suggests that tRNA encoding, ribosomal functionality, and modern metabolic pathways for amino acids and nucleotides developed concurrently, supporting the co-evolution theory of the genetic code [57]. Remarkably, coevolution of anticodon-binding domains and tRNA suggests that the code’s vocabulary expanded from a doublet GC to a triplet GCA and the modern tetraplex GCAU code (Figure 4B), as previously hypothesized [58], [59]. This process involved piecemeal addition of nucleotide stereochemical specificity elements to ancient and expanding tRNA identity elements [29] that took advantage of acceptor-anticodon complementary relationships in the molecules. It is likely that these relationships arose through several segmental duplications in tRNA [28], [60], perhaps mediated by anticodon-binding domain recruitment. We emphasize that code expansion complies with minimizing risks of confusion in the recognition of complementary anticodon-codon pairs that was proposed by Rodin and Rodin [37] and follows closely the associated evolutionary pathways that are constrained by the robustness of the sense-antisense complementarity and its ‘inversion symmetries’ (described in Table S2). The expansion would also comply with patterns of retention of protein structural information [41] uncovered in Figure S5.

### The Biosynthetic Origin of the Code and its Links to Protein Structure and Flexibility

Reduced amino acid alphabets allow formation of fold structures with equal, or even faster, folding rates [61]. Thus, attaining well-packed structural cores in proteins does not require extensive evolutionary optimization and can be achieved with a small amino acid repertoire. We have identified that the inception of the operational code involves catalytic domains with *Group 1* TyrRS, LeuRS and SerRS structures, some of which embed atypical molecular functions. Most notably, SerRS-homologous aaACPLs carry ligase functions resembling those of NRPS aminoacylating modules [42] and TyrRS-homologous CDPSs make use of two aminoacylated tRNAs produced by *Groups 1 and 2* aaRSs to make a range of cyclodipeptide molecules [43] (Figure S6). Thus, archaic aaRS catalytic domains embed structural and functional relics of ligase and peptide biosynthetic functions that could have been responsible for enzymatic production of primordial dipeptides and polypeptides. One prediction of this hypothesis of origin is that primordial dipeptides impacted the amino acid content of emerging peptides and proteins. This prediction was tested by an exhaustive analysis of amino acid composition and sequentially overlapped amino acid dipeptide sequences in 2,384 single-domain proteins of known structure (Figure 5). Remarkably, dipeptides holding *Group 1 and 2* amino acids were enriched in proteins originating before the appearance of anticodon-binding functions and modern genetics. The enriched dipeptides in the ancient proteins were statistically disfavored in loops and their boundaries with regular structures, but favored in boundaries with turns (Figure 6C, Figure 7), which are believed to be ancient (e.g.; [13], [18]) and impact expansions in the most ancient codon exchange graph (Figure S5). Loops are non-regular compact structures (without fixed internal H-bonding) endowed with conformational diversity and generally holding molecular functions [62]. The unanticipated conclusion is that the development of the standard genetic code endowed ancient proteins with flexible unstructured regions that helped optimize catalysis and allosteric regulation through diverse structural topologies. The cryptic connection between dipeptide makeup and code specificity is therefore established: the genetic code originated as an exacting mechanism that linked structure to protein-nucleic acid interactions responsible for archaic peptide biosynthesis. This explanation provides clear evolutionary drivers for genetic code evolution: improved folding for more stable and accessible proteins and development of conformational flexibility and molecular specificities for cellular stability and persistence [7]. In this regard, we note two recent reports of significance. First, phylogenomic analysis of non-local intermolecular contacts (contact order) revealed an early pervasive trend to enhance folding speed during protein evolution [63]. Since fast folding is correlated with flexibility, the trends to fold quickly seem directly linked to the rise of flexibility through genetics. Second, the generation of a protein RNA ligase evolved by *in vitro* evolution from a non-catalytic folded scaffold, lost helical regions and replaced them with a long unstructured loop with enhanced conformational dynamics [64].

**Figure 8:**
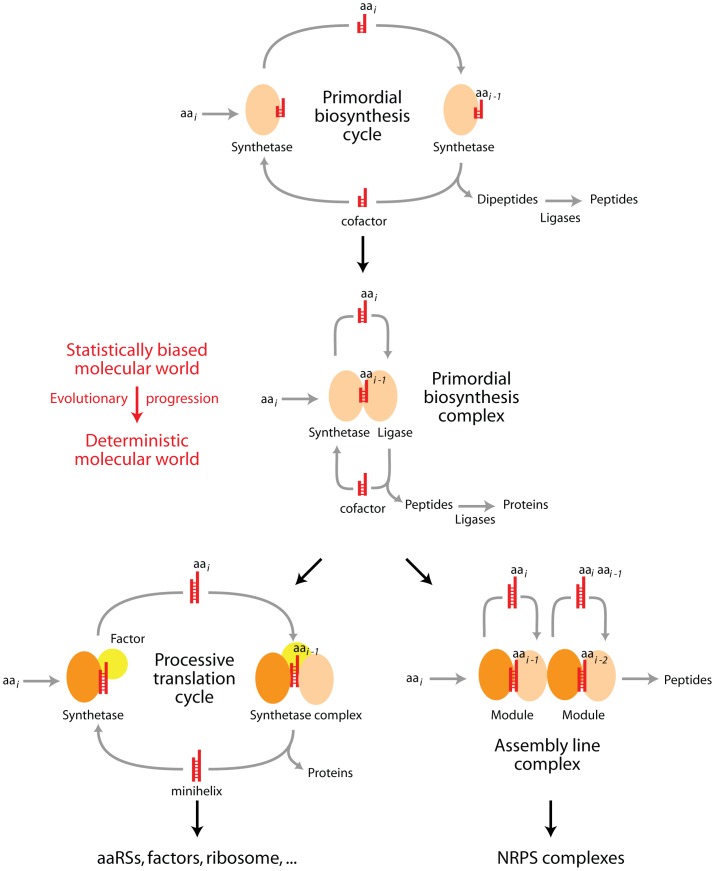
Model of origin and evolution of archaic protein biosynthesis. The flow diagram describes the evolutionary progression of protein biosynthesis and its diversification into ribosome-like processive and NRPS-like assembly-line systems. Translation starts with archaic non-specific synthetases capable of producing dipeptides and small peptides [7]. We assume that these primordial enzymes were originally peptides of less than 60 amino acid residues that emerged from a pool of small peptides (some of them ∼ 25 residues in length and loop-forming) through non-specific condensation reactions. These emergent molecules quickly gained structural properties and stable molecular functions, all of which were initially driven by enhancements of the persistence of emerging cells [7]. The initial synthetases developed the ability to acylate a wide variety of cofactors (4′-phosphopantetheine, CoA, NADP, and related derivatives, and short polynucleotides) in two-step catalytic reactions involving activated intermediates. Peptides could be further ligated into quasi-statistical proteins by the action of non-specific ligase derivatives of the synthetases. It is likely that prebiotic biases in dipeptide makeup resulting from amino acid chemical synthesis [47], [99] and prebiotic peptide formation [48], [49] acted as initial constraints of the emerging quasi-statistical system. In all cases, the quasi-statistical proteins that were formed achieved only Rossmanoid and bundle folded structures, constrained by primitive membranes, and were founders of the most basal fold structures of our phylogenomic timelines [7]. These included P-loop hydrolases and extended or tandem AAA-ATPase mechanoenzymes, oxydoreductases, chaperones and factors. The initial biases were then enhanced by fortuitous contacts between proteins that were beneficial for the primordial cellular system, including the protection of cofactors from degradation. These contacts stabilized protein biosynthesis complexes that facilitated the initial enzymatic activities, and the resulting ensembles behaved very much as modules as the synthetases diversified and enhanced their catalytic toolkit. In some cases they went to produce assembly line complexes similar to modern NRPS systems. In other cases, some modules interacted with polynucleotides and specialized in aminoacylation reactions, leading to modern aaRS functions. Other modules specialized in processive functions leading to the modern ribosome. Polynucleotides gained in some cases folded structures (minihelices, L-shaped conformations) that tuned the make up of interacting protein structures. These initial chains became ancient genomes and important cofactors, and could have also gained functions as nucleic acid replicases, helicases, and ligases. The model that we here propose is fully compatible with a framework that explains the generation of modules and hierarchical structure in biology [100]. Under this framework, modules emerge through two phases of diversification of parts. In the first phase, parts interact weakly and associate diversely. As they diversify and compete, parts interact and these interactions increasingly constrain their structure and associations, leading to modular structures. In the second phase of diversification, variants of the modules and their functions evolve and become new parts for a new cycle of generation of higher-level modules. In our model, parts are emerging proteins and modules are complexes that gain biosynthetic functions. The model highlights the biphasic patterns of diversification of the underlying framework, which we also see unfolding at the amino acid composition level (Fig. 5) and when studying protein flexibility [63].

Structural phylogenomic and protein compositional data therefore supports an ideographic model of primordial protein biosynthesis (Figure 8), in which: (i) early peptides and proteins were initially quasi-statistical ensembles [1] curbed by enzymatic promiscuity, compositional biases and primordial membrane environments [7], (ii) amino acid charging of nucleotide cofactors and ligase specificities in emerging urzymes unfolded dipeptide and polypeptide synthesis without a standard genetic code, benefiting from amino acid exclusion (sieving) mechanisms, (iii) early amino acid charging of acceptor minihelices became modulated by biases in dipeptide makeup, (iv) primordial dipeptide makeup impinged on the outcomes of dipeptide ligation and affected protein folding while structural accretions enhanced primordial aaRS and tRNA specificities; (v) accretion of anticodon recognition domains co-opted interactions for the emerging standard code once primordial tRNA expanded its original structure, interactions and function; and (vi) the expansion of the genetic code remained imprinted in aaRS-tRNA coevolutionary patterns, which we can mine by phylogenomic reconstruction.

The principle of continuity dictates that proteins must emerge from prior states in a continuous evolutionary process. Historically, these prior states most likely involved dipeptides and small polypeptides with limited ordered structure, followed by larger peptides (with ∼25–60 residues) capable of forming loops and van der Waals locks, and then proteins smaller than the size of an average compact domain (∼100 residues in length) [65]. This progression could have occurred (or is occurring) at different rates, enabling different emergence scenarios. Fast progressions create useful domain structures that coexist with less structured prior forms; slow progressions favor gradual and sequential build-up of each hierarchical level of molecular structure. Mapping of highly conserved ‘elementary functional loop’ (EFL) motifs associated with loops and molecular functions [17] to the very first 54 FFs responsible for metabolic expansions and translation [7] suggest a mode of relatively fast progression (K. Caetano-Anollés and G. Caetano-Anollés, ms. in preparation). Thus, it is likely that primordial synthetases arose quickly in the emergent protein world from the pool of quasi-statistical dipeptides and peptides, and that their size and structure was comparable to single-domain aaRS urzymes that have been recently engineered [51], [52], or at least to substantial subdomains of these enzymes. The conformational and compositional diversity of these archaic synthetases must have been limited enough to guarantee maintenance of catalytic and recognition sites, but evolvable enough to mature into ‘ligase’ forms and later on into more complex translation machinery. In this regard, one main assumption is that the FF domain level defined by SCOP captures the very early stages of protein evolution, even if prior states of structural granularity are not studied. There is ample evidence that history is preserved at different levels of the structural hierarchy despite of differences in their evolutionary dynamics [5]. Lower levels of structural abstraction approaching protein sequence are known to change in evolution at faster pace than those at higher levels approaching the fold. In fact, there appears to be a correlation between rates of change and levels of structure [5]. However, small and rare structural features of evolutionary conservation (e.g. ancient motifs in loop regions, conserved active sites and Pfam motifs) are preserved at lower levels while larger and more global conserved attributes (e.g., protein domains, folds, homomeric complexes) are made evident at higher structural levels. This may be again a consequence of the dynamics of generation of structure, which enables the cooption of modules that are generated quickly at lower levels of structural abstraction to build, at lower pace, larger modules at higher levels [46]. As a consequence, evolutionary signal in the more dynamic loops is still captured by the slowly unfolding FF domain structures.

If indeed, the rates of generation of domains from prior forms were high, what mechanisms could have enhanced domain innovation in the emerging protein world? In our model, we propose that the initial drivers for the production of early peptides and proteins were membrane and vesicle stability, a concept that was previously elaborated [7]. The hydrophobic nature of most ancient and most frequent amino acids, as measured by a simple hydropathy scale in our study, supports this putative link. We contend that early biochemistries arose from coevolution of emerging proteins with many molecular species, not just amphiphilic molecules [66]. These inter-molecular interactions enhanced the persistence of the emerging cellular structures and curbed early biochemical evolution. Since small integral peptides tend to self-aggregate into α-helical bundle structures (reviewed in [7]), emerging globular and membrane-tethered ligases could have joined C- or N-terminal tails protruding toward the inner lumen of the primordial vesicles and formed larger membrane-tethered peptides and proteins. These membrane-tethered tails could have provided unique opportunities for combinatorial enhancements of the makeup of loops and globular structure, acting effectively as initial cauldrons of loop combinatorics.

### Cooption as a Primordial Evolutionary Force

A late development of genetics occurring after the inception of protein-catalyzed peptide bond synthesis requires that information in primordial peptides and proteins that preceded mature forms of dipeptidases and ligases be coopted by the emerging genetic system [7], [66]. Specifically, the primordial structural cores of the four FFs that appeared prior to catalytic domains of aaRSs [6], [7] must be encoded by the emerging aaRS-based genetic system in interaction with primordial tRNA or cofactors. In other words, useful links between amino acid composition and structure that benefit molecular functions must be coordinately registered as nucleic acid cofactor-linked specificities by the emerging dipeptidase and ligases, making those same structures accessible to the cells. This can be perceived as a ‘chicken-and-egg’ dilemma. However, if early peptides were quasi-statistical ensembles curbed by strong biases in amino acid and dipeptide makeup, as previous experimental evidence suggests [49], then these biases would be carried on (coopted) by the emerging nucleic acid-protein recognition mechanisms that were being embedded in the unfolding translation system. This would have ensured that the emergent coding rules would preserve the primordial structural biases of the initial four FFs that were existent at that time. Again, a coevolution scenario makes sure that information in prior forms (dipeptides, primordial loops, peptides) is faithfully passed to the emerging domains by directly encoding those biases in the makeup of the emerging ‘genetic’ mediator. This explanation aligns with remarkable evidence of memory [66], including the existence of organisms with faulty aaRS enzymes and ‘statistical’ proteomes that are still able to maintain organism viability despite loss of crucial hydrolytic editing abilities (e.g., [50]) and the observation that protein structure can be efficiently achieved with a limited amino acid repertoire [61]. In fact, a recent study of the effect of mistranslation on the codons of amino acids that bind or do not bind to small molecular ligands support the robustness to genotypic change of functions linked to active sites in proteins (phenotypes) [67]. These findings provide further support to a long line of evidence that suggest that natural selection for error mitigation can affect the robustness of more elementary units of molecular structure (our prior forms) at the translation level (e.g., [68]).

### Explanatory Power: Assumptions and Predictions

Our ‘bold’ conjecture for the origin of genetics departs from standard ‘world’ paradigms dominated by ancient RNA-based replicators, which we consider unrealistic and incapable of withstanding severity of test [66]. Supported by retrodiction and reciprocal illumination [33], [34], the explanatory power *E* of our conjecture *h*, which is derived from phylogenies and timelines, explains evidence *e* given background knowledge *b* according to Popper’s formula:
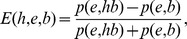
where *p(e,hb)* denotes the probability (and likelihood) of *e* given both *h* and *b*, and *p(e,b)* the probability of *e* given *b*.

Here, hypothesis *h* is a conjunction ensemble that includes phylogenomic trees, timelines of aaRS domains and tRNA structures, minimum number of additional steps required to force hypotheses of monophyly, and statistical links between amino acid and dipeptide compositions associated with protein secondary structure and historical statements, all of which explain the actual patterns and processes of origin.

Evidence *e* include matrices of FF structural homologies and tRNA substructural features from information in genomes and molecules, biases in amino acid and dipeptide compositions of FFs, genetic code complementarity and degeneracy and mappings to amino acid physicochemical properties, and functional and structural evidence from biochemistry and structural biology that support amino acid charging specificities. A number of assumptions relate to *e* that we want to make explicit: (i) Structural homologies at FF level and tRNA substructure level rely on the ability of hidden Markov models (HMMs) to encapsulate the structural design of molecules even in the presence of continuities in the space of protein and RNA sequence [5], [69]. This requires establishing optimal *E* cutoff values capable of maximizing consistency and information content of thousands of phylogenetic characters, optimizing methods of topological correspondence, and increasing the machine learning power of HMMs of structural recognition. (ii) The sampling of the world of proteins is sufficiently trusted to draw reliable evolutionary conclusions, even in the presence of biases in the choice of organisms with genomes that have been sequenced and the overrepresentation of microbes with parasitic lifestyles [5]. (iii) Equating biases in amino acid and dipeptide compositions to ancestral dipeptide compositions require that CDPSs and aaACPLs doppelgängers of archaic dipeptidases and ligases truly encapsulate our interpretation of ancient functions [7]. This necessitates confidence in the statistical approaches and biological rationale we use to link history, structure and amino acid composition, especially because our understanding of the operational code and archaic CDPSs and aaRSs is limited and mostly based on interactions with tRNA or RNA analogs, and not with other cofactors.

Background knowledge *b* includes support for character transformation series defined by the evolutionary model of growth of molecular structures and hypotheses of character polarization (rooting) (*see* Materials and Methods), the existence of a molecular clock of domain structures that link phylogenomic evidence with evidence from fossils and geochemical, biochemical, and biomarker data [13], minimization of risks of confusion in the recognition of complementary anticodon-codon pairs [37], reduction of genetic code ambiguity [36], and compliance with evidence from biochemistry, molecular biology and genomics that we will not make here explicit. Since parsimony and likelihood converge under the ‘no common mechanism’ (discussed in [33]) and other more realistic evolutionary scenarios [70], reciprocal illumination in parsimony increases *E*, especially when *h* strengthens from evidence supporting *b* within the Popperian corroboration framework [34]. A number of important assumptions relate to *b*, of which we will mention only two: (i) the popularity of domain structures (abundance) increases in time at global levels in the protein world, through gene duplication, *de novo* creation, cooption and rearrangements, as a general consequence of mutational diffusion of macromolecules in sequence space [49]; and (ii) structure evolves by reducing the number of possible conformations that can form, which can be many, and by increasing the stability and average life of only a few in a process called ‘structural canalization’ [69]. Increases in molecular abundance with time ensure innovations are not easily lost and structural canalization of conformers enable durable molecular functions.

Important predictions can be made from *h*, some of which we have already discussed, including the effect of compositional biases in mutational robustness. In particular, history of dipeptides and other elementary units of molecular structure are expected to manifest in fast-evolving genomic sequences responsible for *de novo* creation of genes [71], [72] as well as in the protracted history of mature protein-encoding genes. Phylogenetic analysis should be able to make that history evident.

## Materials and Methods

### Structural Phylogenomic Analysis

In this study we mapped the evolution of aaRS domains in a published evolutionary timeline of domain appearance at fold family (FF) level of structural abstraction [6], [7]. This timeline was selected for a number of reasons: FFs generally provide structures with unambiguous assignments of molecular functions, the timeline is well annotated, and results can be benchmarked to a description of the rise of early structures and functions [7]. The timeline was derived from a phylogenomic tree of 2,397 FF structures (out of 3,464 defined by the structural classification of proteins (scop) 1.73; [8]) reconstructed from a structural census in the genomes of 420 free-living organisms from all three cellular superkingdoms (FL420). The timeline was for all purposes congruent to a timeline derived from a phylogenomic tree of 3,513 FFs (out of 3,902 defined by scop 1.75) reconstructed from a census of 989 genomes (A989) [14]. In these structural genomic censuses, *hmmscan* of the profile HMMER3 package scans genomic sequences (with probability cutoffs *E* of 10^−4^) against a library of advanced linear HMMs of structural recognition in superfamily [73]. We note that the phylogenomic approach based on structure (summarized in the flow diagram of Figure 1A) is impervious to a number of limitations that plague sequence analysis, such as problems of alignment, character independence, inapplicable characters, saturation and taxon sampling [74], and is even robust against uneven sampling of genomes across the three superkingdoms [75].

Despite robust evolutionary trends across phylogenies [5], the exact order of closely positioned FFs can be debatable in phylogenetic reconstructions of trees with thousands of leaves. For this reason, we sub-selected domains that were part of aaRSs and generated rooted trees describing the evolution of only the FFs associated with these enzymes (Figure S1, panel A). Tree reconstructions were carried out using maximum parsimony as optimality criterion and a combined parsimony ratchet as previously described [6], [14]. The trees were rooted by the Lundberg method, which does not impose a requirement of outgroup taxa. Phylogenetic reliability was evaluated by the nonparametric bootstrap method with 1,000 replicates, with resampling size being the same as the number of the genomes sampled, TBR, and maxtrees unrestricted. The structure of phylogenetic signal in the data was tested by the skewness (*g_1_*) of the length distribution of 1,000 random trees [76]. Tree distribution profiles and metrics of skewness indicated strong cladistics structure (p<0.01). Recovered trees were well resolved and had basal topologies that matched those of homologous subtrees in the published trees of FFs. Bootstrap support (BS) values for basal branches were 100 and ranged 56–83 in more derived branches; the very derived regions were variable within the 9 most parsimonious trees that were retained. This indicates topologies provide strong support to phylogenetic statements, with support increasing towards the base of the tree. In a recent study, we also reconstructed trees of aaRS domains [77]. These trees were derived from a census of protein structure in 1,037 genomes that included organisms in the three superkingdoms and viruses. Again, topologies were remarkably consistent. Given these results, we used domain ages obtained from the global published phylogenies to place aaRSs in the timeline along with other domains linked to the ribosome and non-ribosomal protein synthetases (NRPS) that we used as reference (Figure 2). For simplicity, domains are here identified with *concise classification strings* (*ccs*). For example, the catalytic domain of tyrosyl-tRNA synthetase [EC 6.1.1.1] corresponds to the c.26.1.1 FF, in which c represents the protein class (α/β proteins), 26 the F (adenine nucleotide alpha hydrolase-like fold), 1 the FSF (nucleotidylyl transferase superfamily), and 1 the FF (Class I aaRSs, catalytic domain).

The relative age of protein structures (*nd*) was calculated directly from the rooted trees using a script that counts the number of nodes from the root (base) of the tree to each leaf and provides it in a relative zero-to-one scale. These *nd* values take advantage of the highly imbalanced nature of the trees of domain structures, as recently discussed [78]. Tree imbalance in these trees is a natural consequence of a heritable trait [79], the accumulation of domain structures in proteins and proteomes [13], which naturally poises speciation [80]. In fact, the δ-test [81] confirms that imbalance in trees of domains was not the result of the node density artifact and represents a true evolutionary process. Moreover, we find that trees do not follow random or Yule models of speciation, which can be considered to drive the evolution of species [13]. The *nd* values are also good proxy for geological time. The molecular clocks for protein domains at fold (F)(t = –3.802 *nd_F_* +3.814) and fold-superfamily (FSF)(t = –3.831 *nd_FSF_* +3.628)(11) levels were used to calculate the geological ages of selected families (in billions of years; Gya), provided that FFs were the most ancient in each group. We note that extending the clock to FFs showed that domain age continued to be proportional to time but with larger dispersion at high *nd*_FF_ values. Clocks were calibrated with geological ages derived from the study of fossils and geochemical, biochemical, and biomarker data, which are affected by the validity of the assumptions used in each and every one of the supporting studies [13]. We also note that the molecular clock derived from trees of Fs and FSFs is necessarily dependent on the rates of domain discovery and accumulation that could be deviant for some domain structures. These factors could cause departures from a clock, with overdispersion sometimes resulting from changes in foldability and structural stability of domains [63].

### Phylogenetic Analysis of tRNA Structure

The ages of tRNA molecules here used were derived from published timelines of amino acid charging and encoding generated from trees of tRNA structure [28]. The method extracts phylogenetic signatures from structural topology in RNA [22], [23], [28], [29], [82]–[87]. These signatures are drawn from links between secondary structure and conformation, dynamics and adaptation [88]. Geometrical and statistical features of RNA substructures are scored in thousands of molecules and this information is analyzed with modern phylogenetic methods to produce *trees of molecules* and *trees of substructures* that portray the history of the system (molecules) or its component parts (substructures), respectively (Figure 1C). The phylogenetic model automatically roots the trees by assuming conformational stability increases in evolution as structures become canalized. The validity of polarization and rooting depends on the axiomatic component of character transformation, which is supported by considerable evidence and is also falsifiable [87].

Phylogenetic constraint analysis restricts the search for optimal trees of tRNAs to pre-specified topologies [29], [85] and can provide important insights from trees of molecules. Here, the minimum number of additional steps (*S*) required to force groups of tRNA taxa in trees (non-mutually exclusive hypotheses) defined measures of ancestrality of individual groups and were used to build evolutionary timelines of amino acid charging and amino acid encoding. Hypotheses with smaller *S* were considered less affected by recruitment and represented processes that were more ancient. Using this approach, chronologies of amino acid charging and codon discovery were directly derived for isoacceptor (*S*_aac_) and anticodon-specific (*S*_cod_) tRNAs, respectively. The validity of character argumentation and the assumption that groups that require lower number of steps are deemed more ancient was derived from the rooted trees and the model of character polarization [87].

### Finding Coevolutionary Patterns

The ages of protein domain structures at FF level (*nd_FF_*) were plotted against the ages of isoacceptor tRNAs (*S_aac_*) and anticodon-specific tRNAs (*S_cod_*). We found that correlations were significant in all instances (*P*<0.0067). Regression lines unfolded evolutionary timelines of archaic editing functions and anticodon-binding specificities, once editing and other accessory domains were identified. Specifically, catalytic and editing domains appearing before anticodon-binding domains (*nd*_FF_ <0.196) and editing domains for AlaRS, ThrRS and PheRS appearing after that age (during a period in which both the operational and standard genetic code were unfolding) were included in correlation studies. Regression timelines were also compared to a conservative idealized timeline that spans domain age and underweights tRNA evolution (relative rates of structural change in protein domains can be 4.3 times higher than in tRNA). Two groups of aaRSs appearing close together in the idealized timeline are part of well-established superclusters defined by sequence and structural analysis [89], superclusters of Class I LeuRS, MetRS, IleRS and ValRS and Class II SerRS and ProRS (Figure 2B). In this regard, higher *S*_aac_ values of tRNAs interacting with structurally related ProRS, LysRS and MetRS makes them evolutionarily derived when compared to SerRS, a fact that is made explicit by the coevolutionary patterns. A recent sequence-based analysis of SerRS, ProRS and ThrRS shows indeed the late appearance of ThrRS [90]. This observation supports the timeline’s validity and the reuse of domain structures in evolution to unfold different specificities.

### Genetic Code Complementarity and Identity Elements

Putative imprints in the primordial complementarity proposed to exist in the genetic code were borrowed directly from Rodin and Rodin [37]. Nucleotides were defined according to IUPAC-IUB Commission of Biochemical Nomenclature. Identity elements in tRNA were identified by in vitro and in vivo approaches [25]–[27]. Loss of aminoacylation efficiency (L) for identity elements in tRNA were given as L = (k/*K*_m_)_wt_/(k/*K*_m_)_mutant_, with rate constants being either *k*_cat_ or V_max_ [25]. Patterns of conservation in tRNA were derived from tRNAdb and identified in tRNAs using established nucleotide numbers [91].

### Analysis of Protein Sequences and Structures

We analyzed amino acid frequencies in secondary structures for a non-culled and a culled set of protein entries from the Protein Data Bank (PDB) (Figure S1). Secondary structures were assigned using the DSSP program [92]. The non-culled set included 204,531 domain sequences (51,392,487 amino acids) downloaded from the PDB (June 20, 2012). The culled set of high quality PDB entries was selected using the protein sequence-culling server PISCES [93] with the following thresholds: sequence percentage identity, ≤25%; resolution, 0.0–2.5; R-factor, 0.25; sequence length, 40–10,000; exclusion of non-X-ray and Cα-only entries; culling by chain. The culled set included 6,828 sequences (1,654,074 amino acids). Since data set sizes are large (*n* >100), it is appropriate to use a parametric method (ANOVA) to test whether there is significant difference among the frequencies of the three amino acid groups (helix, turn and strand) in every secondary structure.

In order to calculate amino acid frequencies in different FFs, we assigned structures to PDB sequences with the HMMs from superfamily [73]. FF assignments were defined according to the SCOP database [8]. The identity of PDB sequences was set as ≤25% using PISCES. All PDB sequences with two or more FFs or unassigned ranges longer than 30 amino acids were eliminated from the study. In the final PDB sequence set, there were 2,384 sequences covered by 1,475 FFs.

We also examined whether or not each of the 20 amino acids or the 408 possible dipeptides that included 2 ambiguous amino acids, Z and X, was respectively enriched in FFs that were more ancient than the oldest anticodon-binding domain of the timeline (305 sequences appearing earlier than c.51.1.1, *nd_FF_ <*0.2) when compared to the set of 2,384 sequences and 1,475 FFs appearing throughout the timeline (*nd = *0–1). For each of the two sequence sets, we counted the numbers of multiple occurrences of amino acids or dipeptides and then calculated the probability of enrichment of every amino acid or dipeptide that was present in the ancient 305-sequence set using the hypergeometric distribution and the following equation [75]:
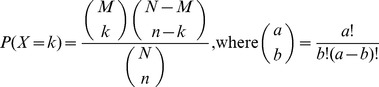



Observed values *M* and *k* indicate the numbers of multiple occurrences of examined amino acids or dipeptides in the 2,384-sequence and the 305-sequence set, respectively. The values *N* and *n* are the numbers of multiple occurrences of all amino acids or dipeptides in the two sequence sets, respectively. The probability *P*(*X = k*) implies the chance that a random variable *X* has *k* multiple occurrences of amino acids or dipeptides for a given amino acid or dipeptide. Referring to the equation and previous literature [94], we calculated *P* values for every amino acid or dipeptide that has *k*/*n* larger than *M*/*N*, and evaluated statistical significance of enrichment of each amino acid or dipeptide in the ancient FFs at 99% confidence levels (*P*<0.01).

### Annotations of Molecular Functions

Molecular functions linked to FFs were annotated using the hierarchical classification of superfamily [73] that assigns seven general functional categories and 50 subcategories to scop IDs [8] based on information in scop, Interpro, Pfam, SwissProt, and literature sources (http://supfam.cs.bris.ac.uk/SUPERFAMILY/function.html). Domain architectures were queried in the PDB database [95] (http://www.rcsb.org/pdb/home/) and annotated using Gene Ontology (GO) [96] (http://www.geneontology.org). GO terms define a vocabulary of molecular functions, biological processes, and cellular components. We examined GO terms linked to molecular functions of translation. Manual annotations also involved queries in the UniProtKB (protein knowledgebase) database (http://www.uniprot.org/) and HMM-based structure assignments. Annotations were mapped onto the domain timeline.

### Structural Alignments

Structural alignments of individual protein structural entries against structural sets were performed using DALI conservation mapping [97] or GANGSTA+ [98]. The DALI server performs pairwise comparisons to PDB90 based on a systematic branch-and-bound search that returns non-overlapping solutions in decreasing order of alignment Z-scores. Selected subsets of structural neighbors were visualized in multiple 3D superpositions for visualization of structural and sequence conservation. Alignments with GANGSTA+, an implementation that uses an advanced non-sequential alignment method with proper assignment of helices and strands in the structure, were against version 1.75 of the ASTRAL40 compendium.

## Supporting Information

Figure S1 **Evolutionary accretion of domains in aaRS enzymes.** A. One of nine most parsimonious phylogenomic tree reconstructions describing the history of the aaRS protein domains analyzed in this study. Terminal leaves are colored according to aaRS class (class I, blue leaves; class II, coral red leaves) and indexed with aaRS domains labeled with *concise classification strings* (*ccs*). The tree matches the corresponding subtree in the global tree of FFs described in the next panel. B. Optimal most parsimonious phylogenomic tree of FFs [177,864 steps; ensemble consistency index (CI) = 0.030; ensemble retention index (RI) = 0.749; g_1_ = −0.070] reconstructed from an analysis of the proteomes of 420 free-living organisms. Terminal leaves are not labeled in the tree since they would not be legible. The Venn diagram shows occurrence of FFs in the three superkingdoms. C. Evolutionary timeline of domain innovation. Domain ages (arrowheads) are mapped along a timeline of FF domain appearance derived from the global phylogenetic tree of FFs. For reference, the timeline is indexed with landmarks derived from domain history [6], [7]. Dashed black lines indicate aaRS history prior to the appearance of the first accessory domain in the structure. The three epochs of the protein world, ‘architectural diversification’, ‘organismal specification’ and ‘superkingdom diversification’ are shaded in green, salmon and yellow, respectively, and are divided into six phases (shade hues) according to Wang et al. [10]. A molecular clock of domain structures places the relative timeline in a geological time scale in billions of years (Gy) [13]. Evolutionary landmark accretion events are indicated with encircled numbers: (1) Catalytic domains have structures with Rossmann-like α/β/α-layered topologies with a central β-sheet flanked by α-helices [53], [101], [102] and can be summarized by the idealized form structure I3_1_ of a periodic table of structures [103]. SerRS, LeuRS, ProRS, LysRS and MetRS structures harbor the most ancient pre-transfer and post-transfer editing functions [2], [27]. The SerRS, LysRS, and MetRS enzymes lack distinct editing domains and probably hydrolyze misactivated amino acids in the active site of the catalytic domain [104]–[106]. Pretransfer editing at the active site has been detected in LeuRS [107] and ProRS [108]. TyrRS lacks internal editing functions [109] but contains a short connecting segment that is homologous to the CP1 editing domain of LeuRS, which harbors species-specific acceptor helix recognition properties [110]. (2) The first editing domain (CP1) has proof reading hydrolase activities that avoid misactivation of Val at frequencies of 1/200. Val mimics the hydrophobic qualities of cognate Ileu and fits in the catalytic pocket of the IleRS enzyme [111]. Similarly, Thr is misactivated by ValRS since both Thr and Val have similar physical and chemical properties and the same barrel structure provides hydrolytic activities. (3) The earliest trans-editing function is provided by D-Tyr-tRNA^Tyr^ deacetylase (DTD). The enzyme hydrolyzes D-Tyr-tRNA^Tyr^ (also D-Trp, D-Ser, D-Asp and D-Glu charged tRNAs) in TyrRSs, which lack internal editing functions [109]. (4) Class IIa anticodon-binding domains of ProRS, GlyRS, ThrRS, HisRS and AlaRS are the first to appear and were closely followed by class Ia (LeuRS, IleuRS, CysRS, MetRS, ValRS and ArgRS) and Ib (GlnRS) anticodon-binding domains. (5) Accretion of aaRS domains, which decorate and enhance the discrimination against non-cognate substrates [112], continues throughout the timeline in parallel with that of ribosomal protein domains. In general, amino acid sieving functions of editing domains appear before recognition of identity elements in the anticodon arm of tRNA by anticodon-binding domains. Accretion encompasses over 2.6 Gy of evolution.
(TIF)

Figure S2 **The origin and evolution of the standard genetic code.** The ancestries of the three anticodon tRNA-aaRS binding expansion groups (A, B and C) were mapped onto a degenerate genetic code table.
(TIF)

Figure S3 **Distribution of age groups of domains with editing (** ***1*** **,** ***2*** **and** ***3*** **) and anticodon-binding (** ***A*** **,** ***B*** **and** ***C*** **) functions, groups in exchange graphs, and active site participation in Venn diagrams of amino acids describing their physicochemical properties.** Venn diagrams show that the origin of amino acid charging in *Group 1* specificities was associated with a polar, turn-inducing and active-site promoting amino acid (Ser) and hydrophobic aromatic (Tyr) and aliphatic (Leu) counterparts. In turn, the start of genetic encoding was associated with small turn-inducing amino acids. We note however that ancient *Groups 1* and *2* domains charge amino acids with low active site-participation frequencies, the only exception being Ser, while *Group 3* exhibits the opposite trend. The origin of the standard genetic code derived from expansion groups A, B and C (Figure S2) was associated with small and hydrophobic amino acids, supporting early protein links to membrane environments [7]. Remarkably, Venn diagrams of exchange graphs show that *Group H* (helix) is uniquely enriched in large hydrophobic and polar amino acids (3 of each) that can be buried and made non-polar by H-bond formation in α−helices and β−strands, respectively [113]. In contrast, amino acids in *Group T* (turn) (6 small, 5 hydrophobic, 5 polar) and *Group E* (strand) (3 small, 5 hydrophobic, 4 polar) are rather balanced in the overall distribution of properties (see Figure S5). However, 75% of amino acids of *Group T* belong to the small category, perhaps necessary because of constraints in turns and bends, and 71% of amino acids of *Group E* are hydrophobic, perhaps necessary to bury strand elements in sandwiched or barrel conformations.
(TIF)

Figure S4 **Analysis of the number of identity elements in cognate tRNA interacting with** ***Groups 1*** **,** ***2*** **and** ***3*** **aaRS domains and their aminoacylation role estimated by loss of aminoacylation efficiency upon mutation.** Note that identity elements associated with *Group 1* domains retain ancestral features of poor specificity. These elements include N73 discriminator base and the N4:N69 base pair of the acceptor stem, which appear to be a diagnostic identity element of aaRSs with ancient editing functions. SerRS, LeuRS, IleRS ValRS and MetRS uniquely recognize the N4:N69 base pair element. The 3′ terminal A76 of tRNA^Leu^ plays an important dual role in aminoacylation and editing of LeuRSs, as well as in other class I aaRS systems. In SerRS, as in other Class II aaRS systems, the interactions with the acceptor stem of tRNA are limited and those with the anticodon loop absent. A recent study reveals that the phosphate backbone of these few identity elements interact with amino acid side chain residues of SerRS in a solvent-related manner through a conserved network of water molecules [114]. This includes G1 and G2 exocyclic oxygen contacts of acceptor tRNA with Phe267 and helical backbone acceptor contacts with ‘loop 2’ Ser151 with Ser156 in positions spanning C69 to C67, which includes the diagnostic element mentioned above. It would be important to determine if these networks of water molecules are exclusive of the very ancient aaRS systems.
(TIF)

Figure S5 **Evolutionary pathways of code expansion and their possible impact on protein structure.** The standard genetic code maps a set of 64 base triplets (codons) to 20 standard amino acids (plus Sec and Pyl for subsets of organisms), and 3 translation stop signals. Zull and Smith [41] showed that the genetic code could be uniquely dissected into three possible sense-antisense codon exchange graphs that retain secondary structure information in proteins. We analyzed the relative frequencies of amino acids belonging to the exchange graphs in regular protein secondary structures, including α-helices (H), β-bridges (B), β-strands (E), 3_10_-helices (G), π-helices (I), turns (T) and bends (S). **A.** Analysis of frequencies of total amino acids belonging to the three exchange graphs (top diagrams), *Group H* (helix; *Leu*, *Lys*, *Phe*, *Glu*, *Gln*), *Group T* (turn; *Ser*, *Pro*, *Thr*, *Ala*, *Arg*, *Cys*, *Gly*, *Trp*), and *Group E* (strand; *Tyr*, *Met*, *Ileu*, *Val*, *Asn*, *Asp*, *His*), in the secondary structures of 6,828 protein domain sequences. Bars headed by the same letters are not significantly different (*P = *0.05) following an ANOVA and the Tukey post-hoc test. The analysis revealed that each group is indeed enriched in amino acids that participate in helix, turn and strand secondary structures, respectively. The non-culled set of 204,531 domain sequences (51,392,487 amino acids) displayed the same global trends (data not shown). Venn diagrams of chemical properties show that *Group H* is enriched in large hydrophobic and polar amino acids that can be buried in α-helices and β-strands, *Group T* in small amino acids necessary for turns and bends, and *Group E* in hydrophobic amino acids necessary to bury strand elements in sandwiched or barrel conformations (Figure S3). **B.** Analysis of frequencies of amino acids in each codon exchange graph dissected according to evolutionary age *Group 1* (*Leu*, *Ser*, *Tyr*), *Group 2* (*Lys*, *Phe*, *Pro*, *Thr*, *Ala*, *Met*, *Ileu*, *Val*) and *Group 3* (*Glu*, *Gln*, *Arg*, *Cys*, *Gly*, *Trp*, *Asn*, *Asp*, *His*) domains. Dissection of exchange graphs into the three domain age groups indeed confirms that *Group T* is consistently overrepresented in turns and bends throughout code history. In turn, *Group H* is overrepresented in domain age *Groups 1* and *3*, while *Groups E* is only overrepresented in *Group 2*. These patterns show the initial importance of helical and turn structure in early protein evolution (*see* [5] for a discussion). This is compatible with the most ancient domain structures of our timelines of domain evolution and their link to primordial membranes [7]. The finding that the most ancient exchange graph (*Group T*) uniquely implements bends and turns throughout genetic code history is highly significant. Sequence motifs that exist in these loop regions of proteins are the most ancient in the protein world [107]. These ‘elementary functional loop’ (EFL) motifs [17] are uniquely associated with the very first domain structures (especially the first 54 FFs; [7]) responsible for metabolic expansions and translation (K. Caetano-Anollés and G. Caetano-Anollés, ms. in preparation). Since ancient *Groups 1* and *2* domains charge amino acids with low active site-participation frequencies (Figure S3), the early inception of helical and turn structure was not in general associated with molecular functions. In contrast, the rise of *Group 3* and the expansion of the standard genetic code in exchange graphs enhanced active site participation and the appearance of molecular functions.
(TIF)

Figure S6 **Structural alignments of amino acid-[acyl-carrier-protein]-ligases (aaACPLs) and cyclodipeptide synthases (CDPSs) to homologous aaRSs using DALI conservation mapping** [**97**] **and structural entries of the Astral compendium.** **A.** RMSD-Z score plots of 601 structural neighbors of aaACPLs (relative to B110957; 3PZC) with Z scores above 2. The closest structural neighbor of aaCPLs (Z = 26.8; RMSD = 2.7 Å) is an unsusual SerRS enzyme from a metanogenic archaeon, *Methanosarcina barkeri* (PDB entry 2CJ9; [115]). The enzyme contains a novel N-terminal domain with increased tRNA variable stem contacts and an active site Zn^2+^ ion-dependent recognition mechanism. aaCPLs, such as B110957 (entry 3PZC), align to the d.104.1.1 catalytic core of the class II SerRS enzyme. **B.** RMSD-Z score plots of 785 structural neighbors of CDPSs (relative to AlbC; 3OQV) with Z scores above 2. The closest neighbors of CDPSs are TyrRSs (Z = 10.0–10.9, RMSD = 3.2–3.8 Å) from archaeal microbes. We note that lower Z scores recover matches to aaRSs from bacteria and eukaryotes, a tendency that supports the ancient origins of the structures. In all cases, CDPSs align to the c.26.1.1 catalytic core of class Ic enzymes but lack ATP binding sites. **C.** Structural comparisons of AlbC CDPS to the protein folds in ASTRAL using an advanced algorithmic implementation, GANGSTA+ [90], displayed as a diagram that shows individual RMSD for nonsequential structural alignments plotted against the number of aligned residues. A total of 10,121 structural alignments, 37 of which had more than 50% amino acid residues matching the structure and involved more than 20 aligned residues. A structural model of structural alignment of AlbC (entry 3OQV; red structure) to its best match, an archaeal TyrRS from *Methanocaldococcus jannaschii* (1J1U), is displayed (RMSD = 3.3 Å; aligned residues = 151; % aligned residues = 71%). Overall results show that the structural cores and crucial sequence sites that contact ligands are highly conserved (across a *Z*-score structural similarity range of 40). Thus, functions of aaACPL and CDPS structures appear to be relics and founders of archaic *Group 1 *aaRS biosynthetic activities.
(TIF)

Figure S7 **The frequencies of amino acids specified by** ***Groups 1, 2*** **and** ***3*** **aaRS structures in 2,384 protein sequences (1,475 FFs) were mapped along the evolutionary timeline of FFs (scatter chart) and studied for central tendencies (box-and-whisker plot; symbols indicate means and lines indicate medians).**
(TIF)

Figure S8 **Models of origin of mirror modes of tRNA acceptor stem recognition by aaRSs.** The class I FF domain appears in the timeline concurrently with the GP-binding domain of elongation and initiation factors, the G protein domain (c.37.1.8), at *nd*_FF_ = 0.020. The class II FF domain appears immediately after at *nd*_FF_ = 0.024. The almost concurrent emergence of domains necessary for tRNA aminoacylation and for the formation of ternary complexes with tRNA and other proteins is striking. The finding fulfills coevolutionary assumptions of three non-mutually exclusive models: A. *Complementarity of ancient genes reflects complementary aaRS interactions:* The existence of mirror modes of tRNA acceptor stem recognition has been suggested to reflect complementarity (head-to-tail) of ancient aaRS genes [46], [47], [116]. In this influential model, the sense-antisense reading of strand symmetric genes in ancient RNA molecules (primordial genomes?) triggered the emergence of the genetic code. To fulfill sense and antisense alignment between class I and II motif sequences, an intervening sequence defining a connecting peptide 1 (CP1) module in class I enzymes and an insertion domain and Motif 3 in class I enzymes, which are more recent evolutionary additions (see below), must be removed. Remarkably, the recent construction of minimal catalytic domains (urzymes) of Class I and Class II enzymes showed that they bind ATP quite tightly and that they have reduced affinity for cognate amino acids [51], [52]. Removal of CP1 in class I aaRSs that nests editing domains or the insertion sequence and Motif 3 in class II aaRSs do not abolish catalytic activities. While intervening regions and additional motifs and domains may enhance amino acid specificity, the reconstructed fragments exhibit ∼9 out of ∼14 orders of magnitude in enzymatic rate acceleration. B. *Complementary aaRSs protected primordial tRNA from degradation*: Mirror recognition of tRNA by complementary aaRSs could have protected primordial tRNA from destruction by nucleases and phosphate bond-cleaving metal ions [1]. Modeling suggests aaRSs can bind simultaneously to opposite sides of the tRNA acceptor stems if they are complementary [117], [118]. C. *Recognition resulted from aaRS-driven polypeptide biosynthesis:* aaRSs could have acted in concert with other aaRSs and translation factors (Figure 7) to perform non-coded polypeptide biosynthesis [7] given that their structures can also form peptide bonds, acting for example as CDPSs [43], [119]–[121], or can aminoacylate 4′-phosphopantheteinyl and other prostetic groups of carrier proteins, acting as aaACPLs [42], a function that is typical of NRPS biosynthetic modules. It is noteworthy that CDPSs are tRNA-dependent peptide bond-forming enzymes responsible for a wide variety of diketopiperazine-related natural products [43], [119]–[121]. CDPSs make use of two aminoacylated tRNAs (aa-tRNA) produced by *Groups 1 and 2* aaRS domains to make the two peptide bonds needed for a range of cyclodipeptide molecules (cFL, cFF, cLL, cYY, etc). For the Rv2275 enzyme that produces cYY [120], catalysis occurs with the initial binding of Tyr-tRNA^Tyr,^ which is followed by nucleophilic attack by a Ser^88^ hydroxyl group of the enzyme on the ester carbonyl, which produces a covalently tyrosinoylated enzyme and free Tyr-tRNA. A second Tyr-tRNA^Tyr^ binds after a possible rotation of the side chain that holds the tyrosinoylated catalytic residue and a second nucleophilic attack likely produces a YY dipeptide intermediate, which is then subjected to an unknown mechanism of cyclization chemistry. A similar overall mechanism has been dissected for the AlbC enzyme that produces cFL [120]. The aminoacyl moiety of aa-tRNA interacts with the active site pocket in a way that is similar to TyrRS, involving at least one patch of basic residues, and the ping-pong mechanism is confirmed. However, successive attachment of the two Leu residues to the CDPS enzyme that produces cLL has been also identified [121]. Given sterochemical properties and biochemical activities, protein urzymes could have aminoacylated prostetic groups and biosynthesized peptides, established stereochemical interactions with nucleic acid cofactors, and at the same time protected emerging nucleic acids from degradation, leaving imprints of these activities in the sequence and structures of present day aaRSs. We note that this emergence still vetoes ‘foresight evolution’ (*sensu* [122]), allowing emerging proteins and nucleic acids to coevolve and capture mutually beneficial improvements of ‘molecular encoding’ into their make-up. It does not however explain how emerging proteins and RNA molecules ‘remembered’ change and improvements during very early stages of emergence. While this is not the subject of this contribution, a proposal has been presented elsewhere [7].
(TIF)

Table S1 **The** ***viz-a-viz*** **representation of the genetic code that puts complementary codes head-to-head with each other.** Light gray and black cells mark the two modes of tRNA recognition from the minor and major sides of the acceptor stem of tRNA, respectively. Numbers 1, 2 and 3 denote codon positions and N2 the consensus of the second nucleotide from the 5′ end of the acceptor arm of tRNA. Shades denote the relative age of the ‘operational code’ (taken from Fig. 2) from dark red (ancient) to dark green (recent).
(PDF)

Table S2 **Risks of confusion of complementary anticodons (see Table S1) under four scenarios of aaRS-tRNA recognition of Rodin and Rodin** [**37**] **and their relative ages (** **Fig. 3** **).**
(PDF)

Table S3 **Dipeptide sequences enriched in ancient domains.** Dipeptides are identified by participating amino acids using one-letter codes and listed together with statistical significance values of enrichment (p) and subsets specified by intervening amino acids corresponding to *Group 1*, *2* and *3* domain structures (one of 9 subsets possible).
(PDF)
