# Supplementary figures and images for: Structural Phylogenomics Retrodicts the Origin of the Genetic Code and Uncovers the Evolutionary Impact of Protein Flexibility
Source: PLoS One. 2013 Aug 21;8(8):e72225. doi: 10.1371/journal.pone.0072225 (PMC3749098; doi:10.1371/journal.pone.0072225)

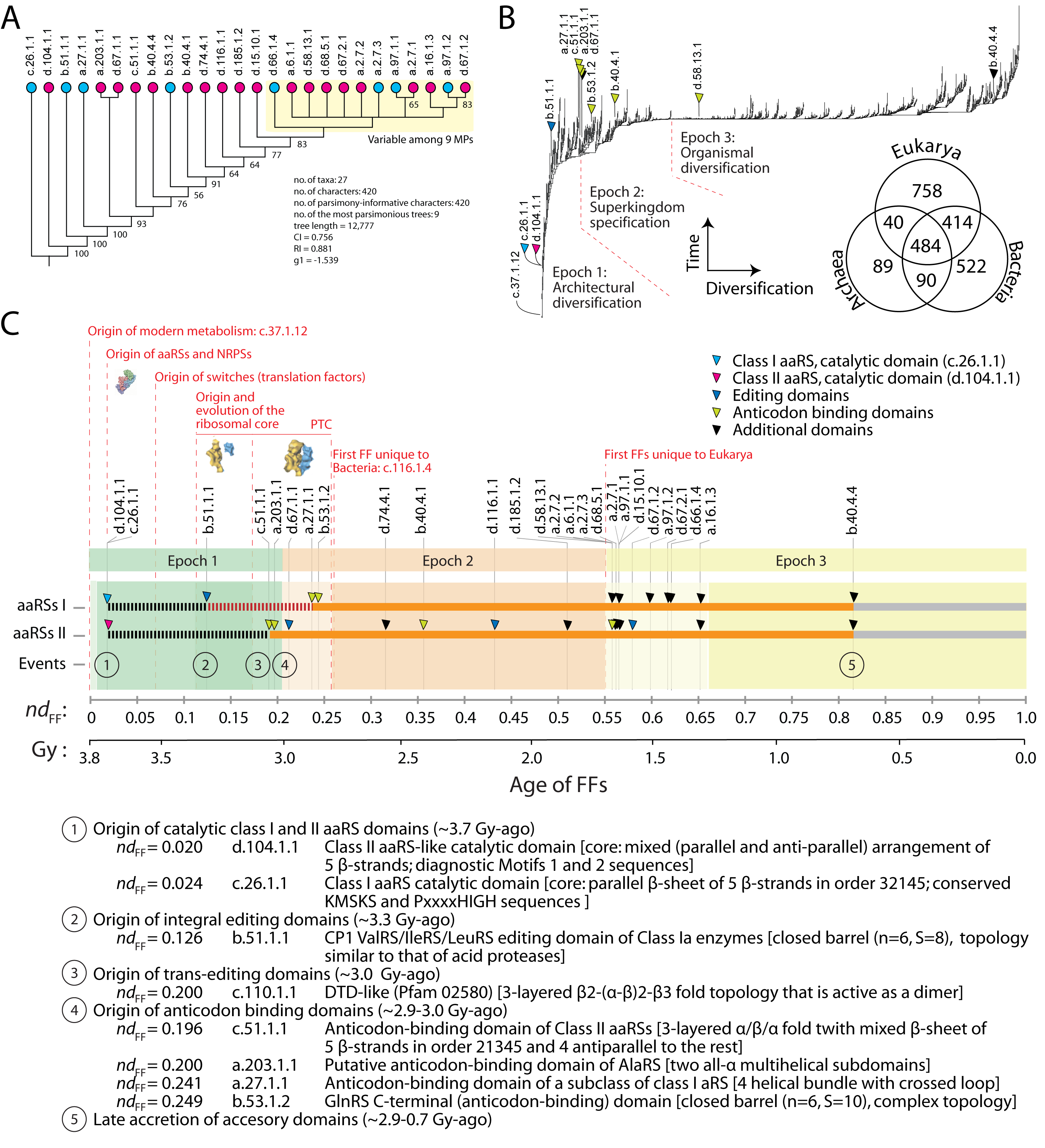

Supplement: Figure S1 — Evolutionary accretion of domains in aaRS enzymes. A. One of nine most parsimonious phylogenomic tree reconstructions describing the history of the aaRS protein domains analyzed in this study. Terminal leaves are colored according to aaRS class (class I, blue leaves; class II, coral red leaves) and indexed with aaRS domains labeled with concise classification strings (ccs). The tree matches the corresponding subtree in the global tree of FFs described in the next panel. B. Optimal most parsimonious phylogenomic tree of FFs [177,864 steps; ensemble consistency index (CI) = 0.030; ensemble retention index (RI) = 0.749; g1 = −0.070] reconstructed from an analysis of the proteomes of 420 free-living organisms. Terminal leaves are not labeled in the tree since they would not be legible. The Venn diagram shows occurrence of FFs in the three superkingdoms. C. Evolutionary timeline of domain innovation. Domain ages (arrowheads) are mapped along a timeline of FF domain appearance derived from the global phylogenetic tree of FFs. For reference, the timeline is indexed with landmarks derived from domain history [6], [7]. Dashed black lines indicate aaRS history prior to the appearance of the first accessory domain in the structure. The three epochs of the protein world, ‘architectural diversification’, ‘organismal specification’ and ‘superkingdom diversification’ are shaded in green, salmon and yellow, respectively, and are divided into six phases (shade hues) according to Wang et al. [10]. A molecular clock of domain structures places the relative timeline in a geological time scale in billions of years (Gy) [13]. Evolutionary landmark accretion events are indicated with encircled numbers: (1) Catalytic domains have structures with Rossmann-like α/β/α-layered topologies with a central β-sheet flanked by α-helices [53], [101], [102] and can be summarized by the idealized form structure I31 of a periodic table of structures [103]. SerRS, LeuRS, ProRS, LysRS and MetRS structures [file pone.0072225.s001.tif]

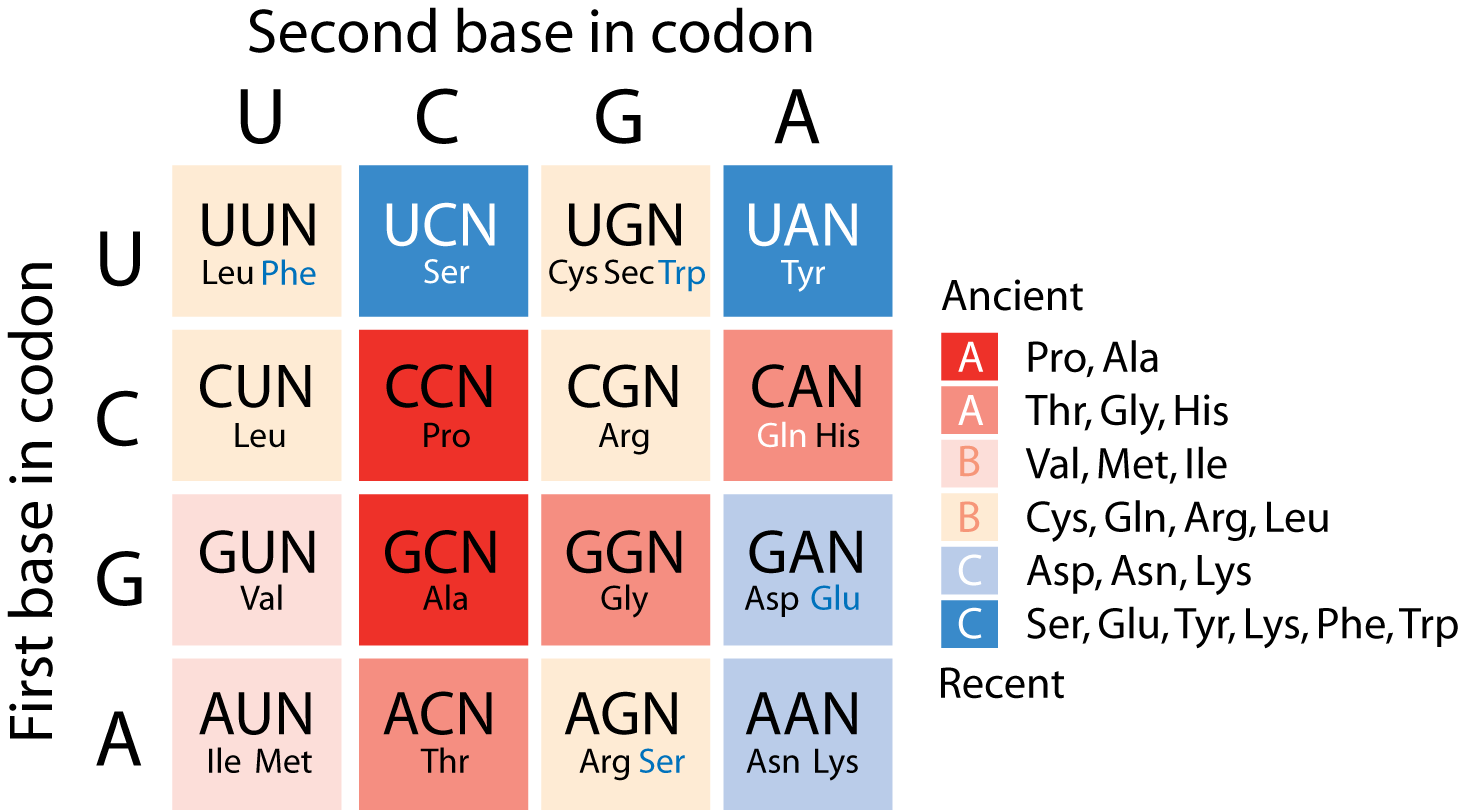

Supplement: Figure S2 — The origin and evolution of the standard genetic code. The ancestries of the three anticodon tRNA-aaRS binding expansion groups (A, B and C) were mapped onto a degenerate genetic code table. (TIF) [file pone.0072225.s002.tif]

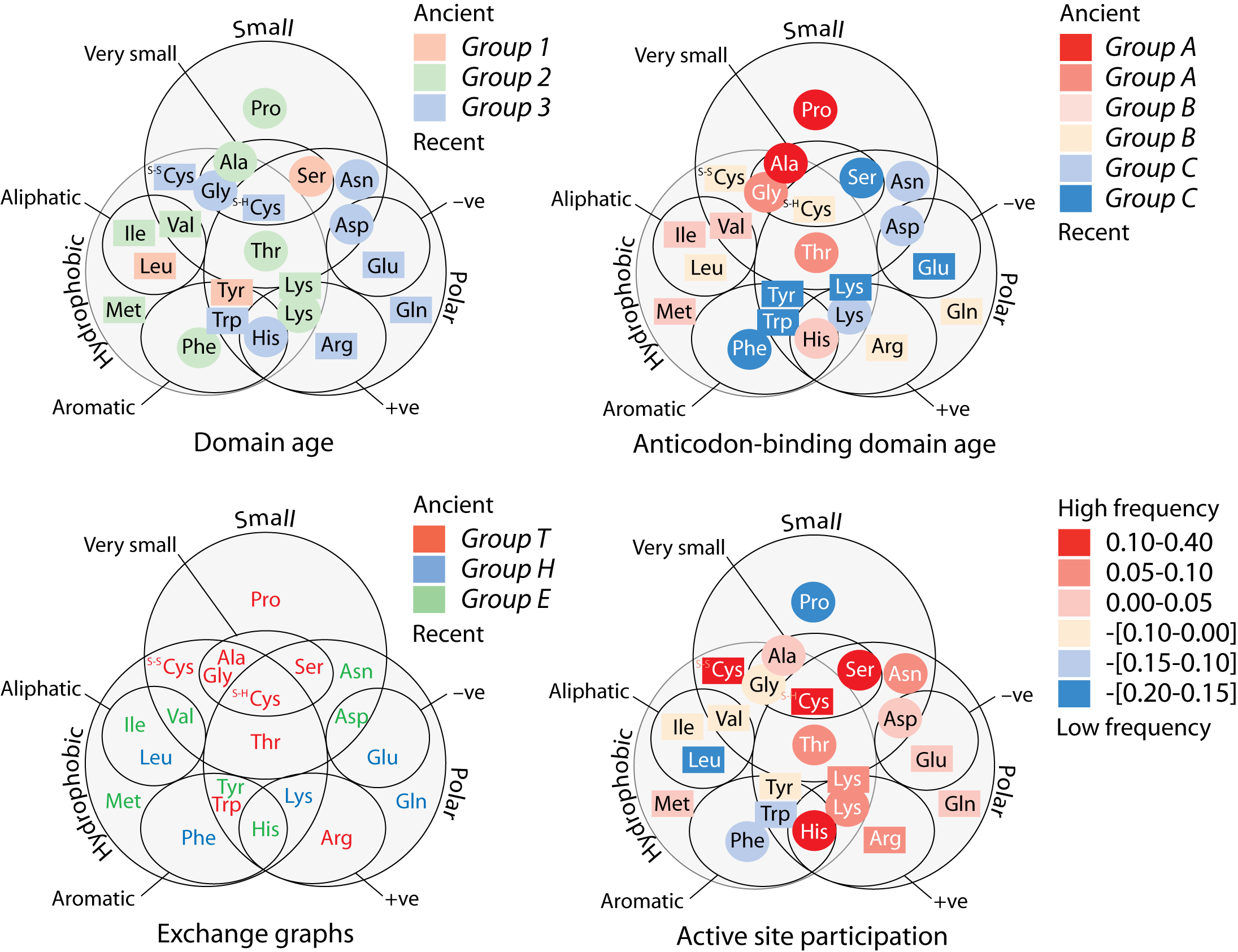

Supplement: Figure S3 — Distribution of age groups of domains with editing ( 1 , 2 and 3 ) and anticodon-binding ( A , B and C ) functions, groups in exchange graphs, and active site participation in Venn diagrams of amino acids describing their physicochemical properties. Venn diagrams show that the origin of amino acid charging in Group 1 specificities was associated with a polar, turn-inducing and active-site promoting amino acid (Ser) and hydrophobic aromatic (Tyr) and aliphatic (Leu) counterparts. In turn, the start of genetic encoding was associated with small turn-inducing amino acids. We note however that ancient Groups 1 and 2 domains charge amino acids with low active site-participation frequencies, the only exception being Ser, while Group 3 exhibits the opposite trend. The origin of the standard genetic code derived from expansion groups A, B and C (Figure S2) was associated with small and hydrophobic amino acids, supporting early protein links to membrane environments [7]. Remarkably, Venn diagrams of exchange graphs show that Group H (helix) is uniquely enriched in large hydrophobic and polar amino acids (3 of each) that can be buried and made non-polar by H-bond formation in α−helices and β−strands, respectively [113]. In contrast, amino acids in Group T (turn) (6 small, 5 hydrophobic, 5 polar) and Group E (strand) (3 small, 5 hydrophobic, 4 polar) are rather balanced in the overall distribution of properties (see Figure S5). However, 75% of amino acids of Group T belong to the small category, perhaps necessary because of constraints in turns and bends, and 71% of amino acids of Group E are hydrophobic, perhaps necessary to bury strand elements in sandwiched or barrel conformations. (TIF) [file pone.0072225.s003.tif]

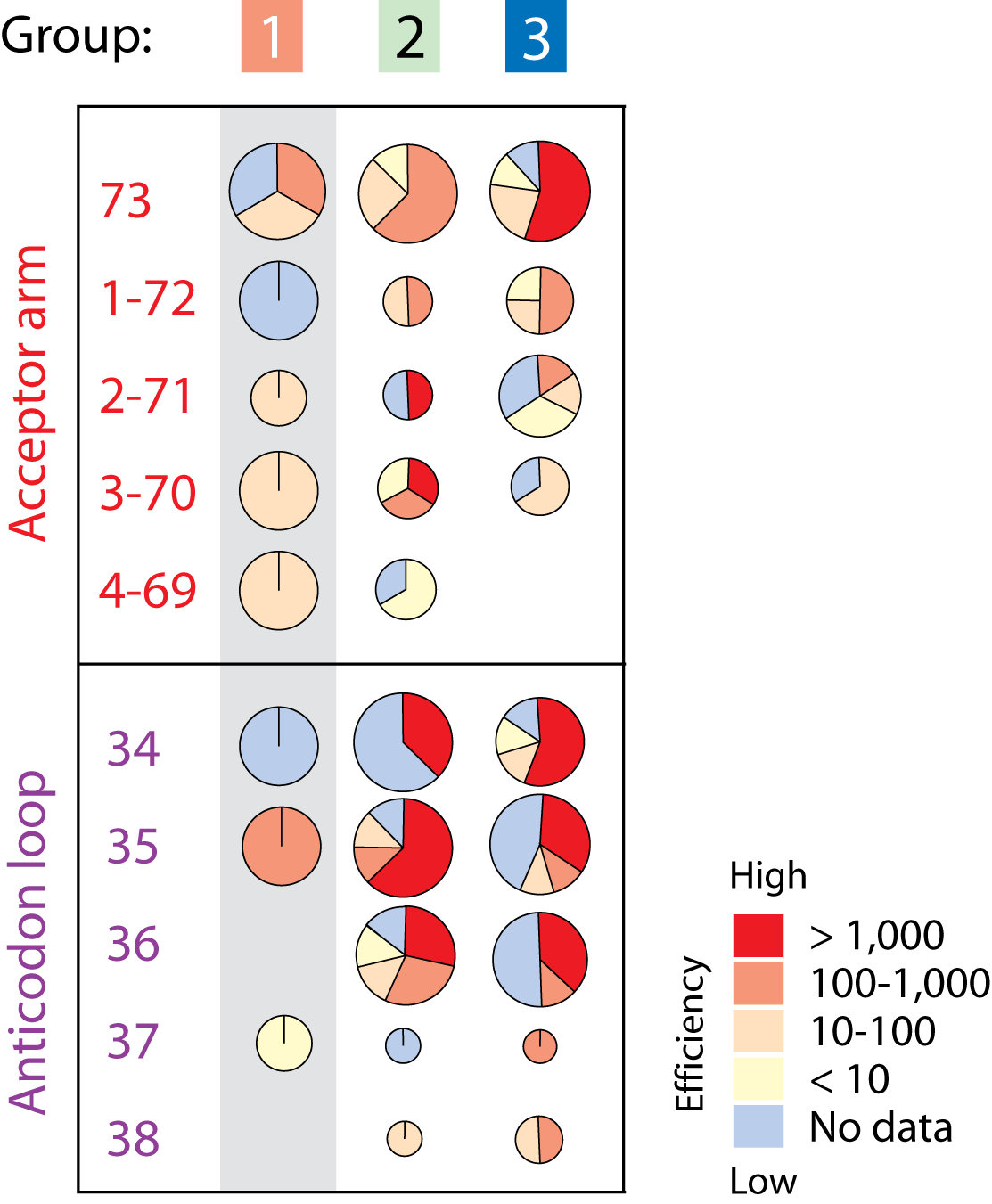

Supplement: Figure S4 — Analysis of the number of identity elements in cognate tRNA interacting with Groups 1 , 2 and 3 aaRS domains and their aminoacylation role estimated by loss of aminoacylation efficiency upon mutation. Note that identity elements associated with Group 1 domains retain ancestral features of poor specificity. These elements include N73 discriminator base and the N4:N69 base pair of the acceptor stem, which appear to be a diagnostic identity element of aaRSs with ancient editing functions. SerRS, LeuRS, IleRS ValRS and MetRS uniquely recognize the N4:N69 base pair element. The 3′ terminal A76 of tRNALeu plays an important dual role in aminoacylation and editing of LeuRSs, as well as in other class I aaRS systems. In SerRS, as in other Class II aaRS systems, the interactions with the acceptor stem of tRNA are limited and those with the anticodon loop absent. A recent study reveals that the phosphate backbone of these few identity elements interact with amino acid side chain residues of SerRS in a solvent-related manner through a conserved network of water molecules [114]. This includes G1 and G2 exocyclic oxygen contacts of acceptor tRNA with Phe267 and helical backbone acceptor contacts with ‘loop 2’ Ser151 with Ser156 in positions spanning C69 to C67, which includes the diagnostic element mentioned above. It would be important to determine if these networks of water molecules are exclusive of the very ancient aaRS systems. (TIF) [file pone.0072225.s004.tif]

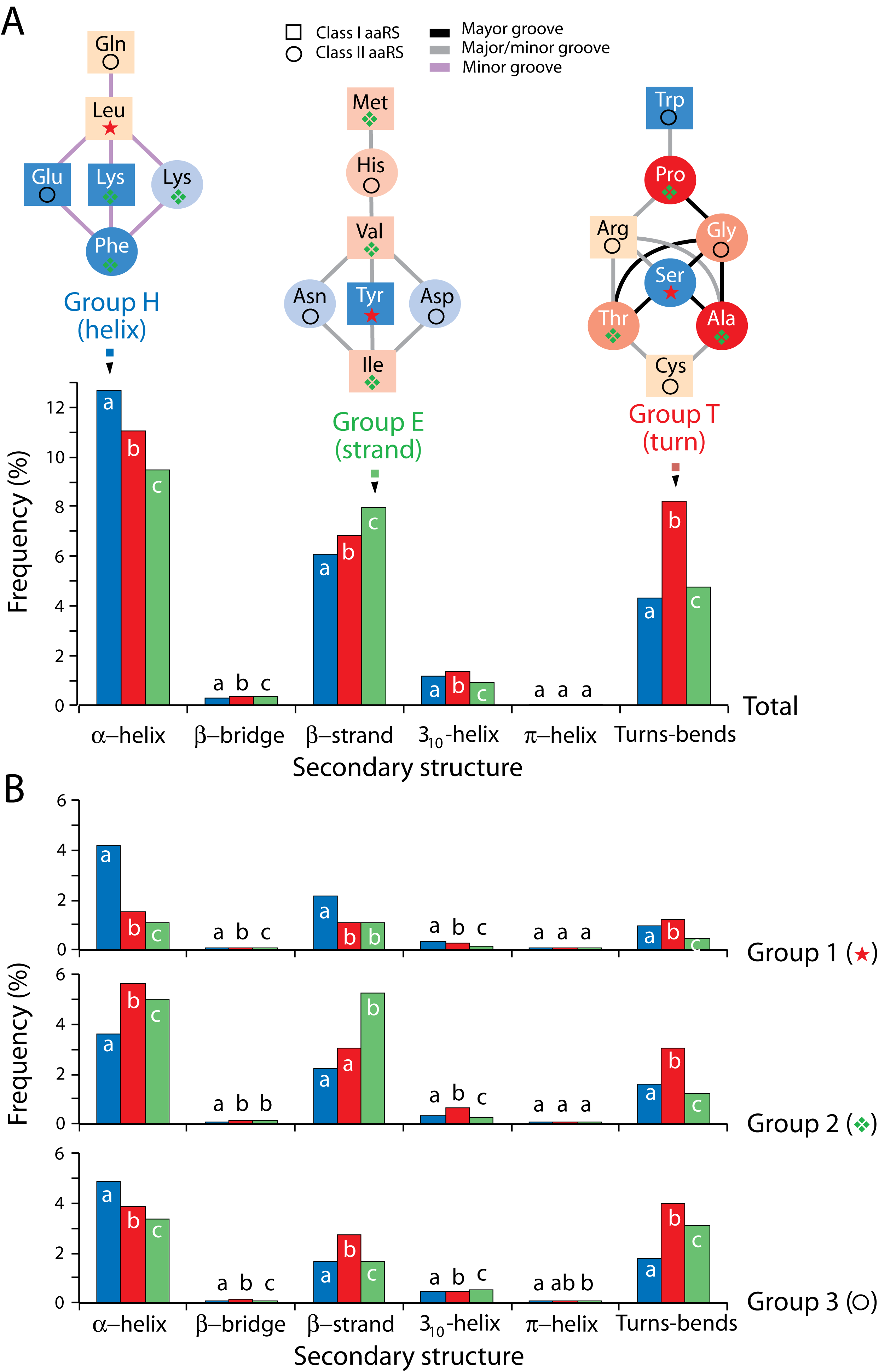

Supplement: Figure S5 — Evolutionary pathways of code expansion and their possible impact on protein structure. The standard genetic code maps a set of 64 base triplets (codons) to 20 standard amino acids (plus Sec and Pyl for subsets of organisms), and 3 translation stop signals. Zull and Smith [41] showed that the genetic code could be uniquely dissected into three possible sense-antisense codon exchange graphs that retain secondary structure information in proteins. We analyzed the relative frequencies of amino acids belonging to the exchange graphs in regular protein secondary structures, including α-helices (H), β-bridges (B), β-strands (E), 310-helices (G), π-helices (I), turns (T) and bends (S). A. Analysis of frequencies of total amino acids belonging to the three exchange graphs (top diagrams), Group H (helix; Leu, Lys, Phe, Glu, Gln), Group T (turn; Ser, Pro, Thr, Ala, Arg, Cys, Gly, Trp), and Group E (strand; Tyr, Met, Ileu, Val, Asn, Asp, His), in the secondary structures of 6,828 protein domain sequences. Bars headed by the same letters are not significantly different (P = 0.05) following an ANOVA and the Tukey post-hoc test. The analysis revealed that each group is indeed enriched in amino acids that participate in helix, turn and strand secondary structures, respectively. The non-culled set of 204,531 domain sequences (51,392,487 amino acids) displayed the same global trends (data not shown). Venn diagrams of chemical properties show that Group H is enriched in large hydrophobic and polar amino acids that can be buried in α-helices and β-strands, Group T in small amino acids necessary for turns and bends, and Group E in hydrophobic amino acids necessary to bury strand elements in sandwiched or barrel conformations (Figure S3). B. Analysis of frequencies of amino acids in each codon exchange graph dissected according to evolutionary age Group 1 (Leu, Ser, Tyr), Group 2 (Lys, Phe, Pro, Thr, Ala, Met, Ileu, Val) and Group 3 (Glu, Gln, Arg, Cys, Gly, Trp, Asn, Asp, His) domains. [file pone.0072225.s005.tif]

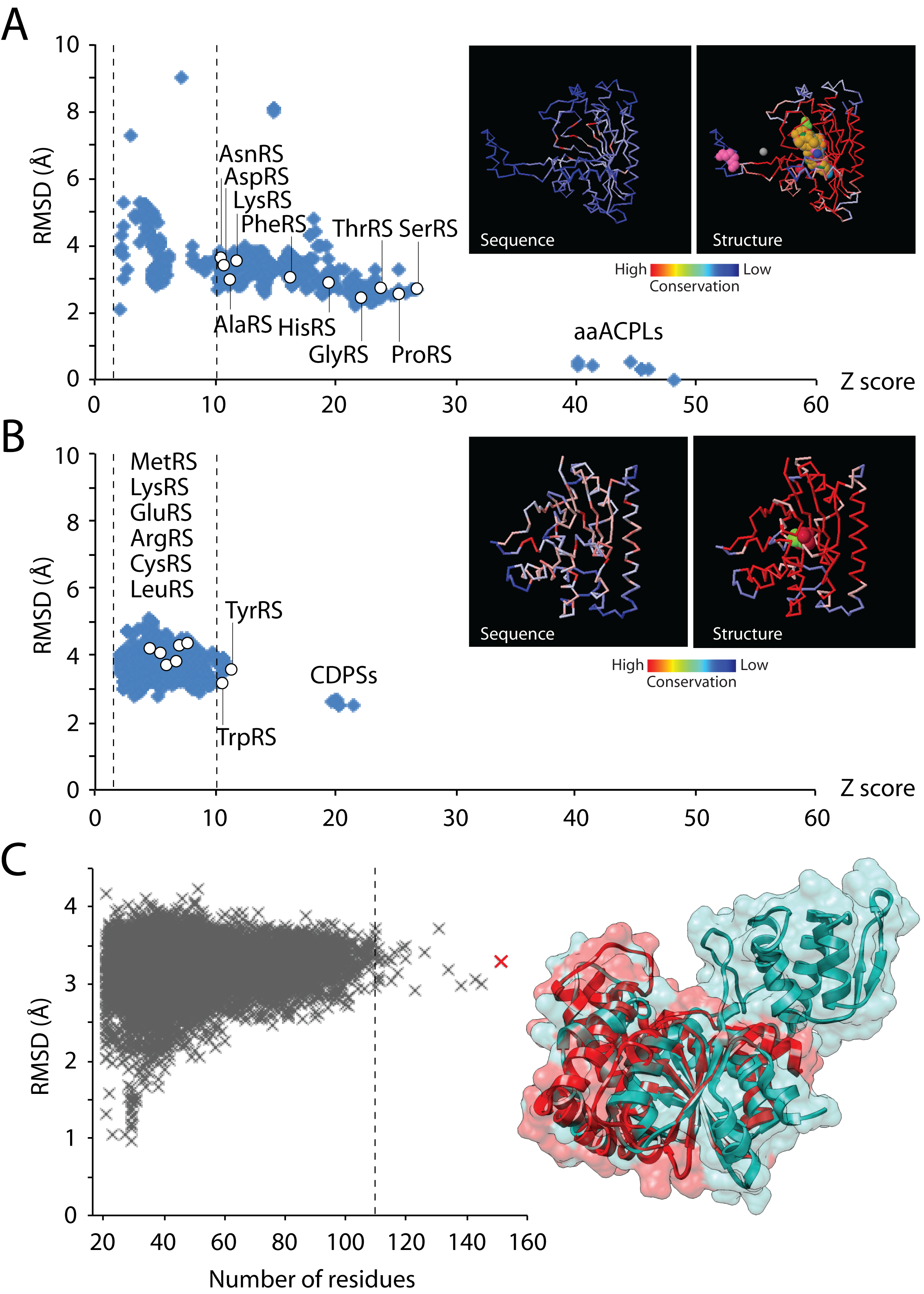

Supplement: Figure S6 — Structural alignments of amino acid-[acyl-carrier-protein]-ligases (aaACPLs) and cyclodipeptide synthases (CDPSs) to homologous aaRSs using DALI conservation mapping [97] and structural entries of the Astral compendium. A. RMSD-Z score plots of 601 structural neighbors of aaACPLs (relative to B110957; 3PZC) with Z scores above 2. The closest structural neighbor of aaCPLs (Z = 26.8; RMSD = 2.7 Å) is an unsusual SerRS enzyme from a metanogenic archaeon, Methanosarcina barkeri (PDB entry 2CJ9; [115]). The enzyme contains a novel N-terminal domain with increased tRNA variable stem contacts and an active site Zn2+ ion-dependent recognition mechanism. aaCPLs, such as B110957 (entry 3PZC), align to the d.104.1.1 catalytic core of the class II SerRS enzyme. B. RMSD-Z score plots of 785 structural neighbors of CDPSs (relative to AlbC; 3OQV) with Z scores above 2. The closest neighbors of CDPSs are TyrRSs (Z = 10.0–10.9, RMSD = 3.2–3.8 Å) from archaeal microbes. We note that lower Z scores recover matches to aaRSs from bacteria and eukaryotes, a tendency that supports the ancient origins of the structures. In all cases, CDPSs align to the c.26.1.1 catalytic core of class Ic enzymes but lack ATP binding sites. C. Structural comparisons of AlbC CDPS to the protein folds in ASTRAL using an advanced algorithmic implementation, GANGSTA+ [90], displayed as a diagram that shows individual RMSD for nonsequential structural alignments plotted against the number of aligned residues. A total of 10,121 structural alignments, 37 of which had more than 50% amino acid residues matching the structure and involved more than 20 aligned residues. A structural model of structural alignment of AlbC (entry 3OQV; red structure) to its best match, an archaeal TyrRS from Methanocaldococcus jannaschii (1J1U), is displayed (RMSD = 3.3 Å; aligned residues = 151; % aligned residues = 71%). Overall results show that the structural cores and crucial sequence sites that contact ligands are highly conserved [file pone.0072225.s006.tif]

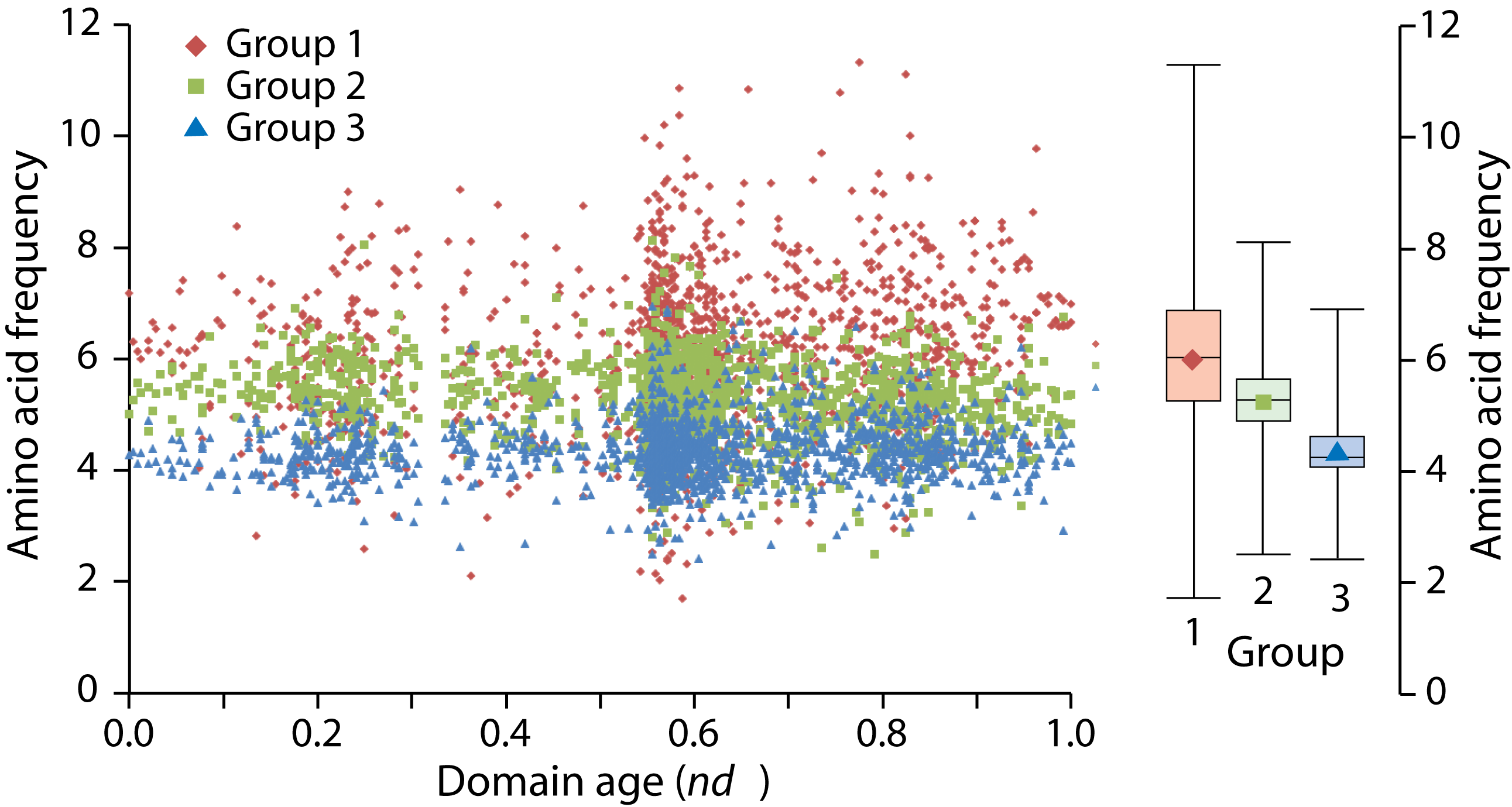

Supplement: Figure S7 — The frequencies of amino acids specified by Groups 1, 2 and 3 aaRS structures in 2,384 protein sequences (1,475 FFs) were mapped along the evolutionary timeline of FFs (scatter chart) and studied for central tendencies (box-and-whisker plot; symbols indicate means and lines indicate medians). (TIF) [file pone.0072225.s007.tif]

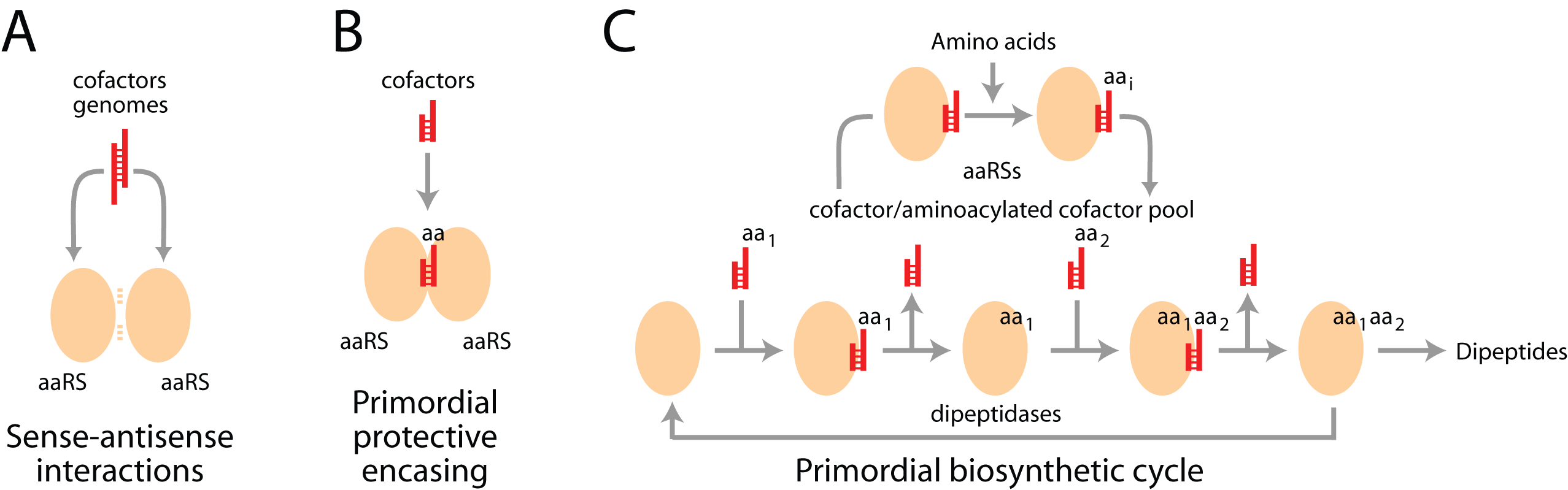

Supplement: Figure S8 — Models of origin of mirror modes of tRNA acceptor stem recognition by aaRSs. The class I FF domain appears in the timeline concurrently with the GP-binding domain of elongation and initiation factors, the G protein domain (c.37.1.8), at nd FF = 0.020. The class II FF domain appears immediately after at nd FF = 0.024. The almost concurrent emergence of domains necessary for tRNA aminoacylation and for the formation of ternary complexes with tRNA and other proteins is striking. The finding fulfills coevolutionary assumptions of three non-mutually exclusive models: A. Complementarity of ancient genes reflects complementary aaRS interactions: The existence of mirror modes of tRNA acceptor stem recognition has been suggested to reflect complementarity (head-to-tail) of ancient aaRS genes [46], [47], [116]. In this influential model, the sense-antisense reading of strand symmetric genes in ancient RNA molecules (primordial genomes?) triggered the emergence of the genetic code. To fulfill sense and antisense alignment between class I and II motif sequences, an intervening sequence defining a connecting peptide 1 (CP1) module in class I enzymes and an insertion domain and Motif 3 in class I enzymes, which are more recent evolutionary additions (see below), must be removed. Remarkably, the recent construction of minimal catalytic domains (urzymes) of Class I and Class II enzymes showed that they bind ATP quite tightly and that they have reduced affinity for cognate amino acids [51], [52]. Removal of CP1 in class I aaRSs that nests editing domains or the insertion sequence and Motif 3 in class II aaRSs do not abolish catalytic activities. While intervening regions and additional motifs and domains may enhance amino acid specificity, the reconstructed fragments exhibit ∼9 out of ∼14 orders of magnitude in enzymatic rate acceleration. B. Complementary aaRSs protected primordial tRNA from degradation: Mirror recognition of tRNA by complementary aaRSs could have protected primord [file pone.0072225.s008.tif]
