# Supplementary material for: Structural Phylogenomics Retrodicts the Origin of the Genetic Code and Uncovers the Evolutionary Impact of Protein Flexibility
Source: PLoS One. 2013 Aug 21;8(8):e72225. doi: 10.1371/journal.pone.0072225 (PMC3749098; doi:10.1371/journal.pone.0072225)
Supplement: Table S1 — The viz-a-viz representation of the genetic code that puts complementary codes head-to-head with each other. Light gray and black cells mark the two modes of tRNA recognition from the minor and major sides of the acceptor stem of tRNA, respectively. Numbers 1, 2 and 3 denote codon positions and N2 the consensus of the second nucleotide from the 5′ end of the acceptor arm of tRNA. Shades denote the relative age of the ‘operational code’ (taken from Fig. 2) from dark red (ancient) to dark green (recent). (PDF) [file pone.0072225.s009.pdf]

Table S1. The *viz-a-viz* representation of the genetic code that puts complementary codes head-to-head with each other. Light gray and black cells mark the two modes of tRNA recognition from the minor and major sides of the acceptor stem of tRNA, respectively. Numbers 1, 2 and 3 denote codon positions and N2 the consensus of the second nucleotide from the 5' end of the acceptor arm of tRNA. Shades denote the relative age of the 'operational code' (taken from Fig. 2) from dark red (ancient) to dark green (recent).

	1	2	3			1	2	3			1	2	3			1	2	3	
	Y	U	N	N2		N	A	R	N2		Y	G	N	N2		N	C	R	N2
Phe	U	U	U	C	Lys	A	A	A	R	Cys	U	G	U	S	Thr	A	C	A	C
Phe	U	U	C	C	Glu	G	A	A	C	Cys	U	G	C	S	Ala	G	C	A	G
Leu	U	U	A	C	Stop Gln	U	A	A	G	Stop Sec	U	G	A	S	Ser	U	C	A	G
Leu	U	U	G	C	Gln	C	A	A	G	Trp	U	G	G	S	Pro	C	C	A	G
Leu	C	U	U	G	Lys	A	A	G	C	Arg	C	G	U	G	Thr	A	C	G	C
Leu	C	U	C	G	Glu	G	A	G	C	Arg	C	G	C	U	Ala	G	C	G	G
Leu	C	U	A	C	Stop Gln	U	A	G	G	Arg	C	G	A	C	Ser	U	C	G	G
Leu	C	U	G	Y	Gln	C	A	G	G	Arg	C	G	G	C	Pro	C	C	G	G

	1	2	3			1	2	3			1	2	3			1	2	3	
	R	U	N	N2		N	A	Y	N2		R	G	N	N2		N	C	Y	N2
Ile	A	U	U	G	Asn	A	A	U	C	Ser (2)	A	G	U	G	Thr	A	C	U	C
Ile	A	U	C	G	Asp	G	A	U	C	Ser (2)	A	G	C	G	Ala	G	C	U	G
Ile	A	U	A	G	Tyr	U	A	U	S	Gly/Ser	A	G	A	G	Ser	U	C	U	G
Met	A	U	G	G	His	C	A	U	Y	Gly/Ser	A	G	G	G	Pro	C	C	U	G
Val	G	U	U	G	Asn	A	A	C	C	Gly	G	G	U	C	Thr	A	C	C	C
Val	G	U	C	G	Asp	G	A	C	C	Gly	G	G	C	C	Ala	G	C	C	G
Val	G	U	A	U	Tyr	U	A	C	S	Gly	G	G	A	C	Ser	U	C	C	G
Val	G	U	G	G	His	C	A	C	Y	Gly	G	G	G	C	Pro	C	C	C	G
