# Supplementary material for: Structural Phylogenomics Retrodicts the Origin of the Genetic Code and Uncovers the Evolutionary Impact of Protein Flexibility
Source: PLoS One. 2013 Aug 21;8(8):e72225. doi: 10.1371/journal.pone.0072225 (PMC3749098; doi:10.1371/journal.pone.0072225)
Supplement: Table S2 — Risks of confusion of complementary anticodons (see Table S1) under four scenarios of aaRS-tRNA recognition of Rodin and Rodin [37] and their relative ages ( Fig. 3 ). (PDF) [file pone.0072225.s010.pdf]

Table S2. Risks of confusion of complementary anticodons (see Table S1) under four scenarios of aaRS-tRNA recognition of Rodin and Rodin [32] and their relative ages (Fig. 3).[a]

Complementarity [avg. age of group]	Pairs of complementary anticodons		5' x 5' mn/mn	5' x 3' mn/MJ	3' x 3' MJ/MJ	3' x 5' MJ/mn	Age of pairs
NAR x YUN [0.70]	Phe (GAA)	Glu (UUC)	+	+	+	+	0.94
	Phe (AAA)	Lys (UUU)	+	+	+	+	1.00
	Leu (CAA)	Gln (UUG)	–	–	–	+	0.54
	Leu (UAA)	Stop (UUA)	–	–	–	+	0.54
	Leu (GAG)	Glu (CUC)	+	+	+	+	0.73
	Leu (AAG)	Lys (CUU)	+	+	+	+	0.79
	Leu (CAG)	Gln (CUG)	–	–	–	+	0.54
	Leu (UAG)	Stop (CUA)	–	–	–	+	0.54
NAY x RUN [0.42]	Ile (GAU)	Asp (AUC)	+	+	+	+	0.52
	Ile (AAU)	Asn (AUU)	+	+	+	+	0.54
	Ile (UAU)	Tyr (AUA)	–	+	–	–	0.64
	Met (CAU)	His (AUG)	–	+	–	–	0.3
	Val (GAC)	Asp (GUC)	+	+	+	+	0.48
	Val (AAC)	Asn (GUU)	+	+	+	+	0.52
	Val (CAC)	His (GUG)	–	+	–	–	0.27
	Val (UAC)	Tyr (GUA)	–	+	–	–	0.61
NCR x YGN [0.45]	Cys (GCA)	Ala (UGC)	–	–	–	+	0.24
	Cys (ACA)	Thr (UGU)	–	–	–	+	0.27
	Trp (CCA)	Pro (UGG)	–	+	–	–	0.54
	Stop (UCA)	Ser (UGA)	–	+	–	–	0.91
	Arg (GCG)	Ala (CGC)	–	–	–	+	0.3
	Arg (ACG)	Thr (CGU)	–	–	–	+	0.33
	Arg (CCG)	Pro (CGG)	–	+	–	–	0.3
	Arg (UCG)	Ser (CGA)	–	+	–	–	0.73
NCY x RGN [0.36]	Ser (GCU)	Ala (AGC)	+	+	+	+	0.42
	Ser (ACU)	Thr (AGU)	+	+	+	+	0.45
	Gly/Ser (CCU)	Pro (AGG)	+	+	+	+	0.42
	Gly/Ser (UCU)	Ser (AGA)	+	+	+	+	0.62
	Gly (GCC)	Ala (GGC)	+	+	+	+	0.12
	Gly (ACC)	Thr (GGU)	+	+	+	+	0.15
	Gly (CCC)	Pro (GGG)	+	+	+	+	0.12
	Gly (UCG)	Ser (GGA)	+	+	+	+	0.54

[a] Plus signs denote anticodon pairs that have no identical tetra(or more)-nucleotides within the loop 3'YU-XYZ-RN5', i.e. they are distinguishable by two aaRSs that recognize the complementary tRNA halves. Minus signs mark indistinguishable cases. For each pair, only a zero- or one base-long shift in one of two directions from the anticodon is allowed. Two simultaneous shifts (one in each anticodon loop) are considered highly unlikely (26). Assigning NGN and NAN anticodons to major (MJ) and minor (mn) groove sides reduces conceivable scenarios of tRNA recognition to only two (shaded cells), and the most parsimonious evolutionary pathway to only one (light green). More details can be found in Rodin and Rodin (26). Ages of pairs of complementary anticodons were calculated directly from the idealized timeline (Fig. 3) in a relative scale from 0 (ancient) to 1 (present), and match predictions (26).
