# Supplementary material for: Structural Phylogenomics Retrodicts the Origin of the Genetic Code and Uncovers the Evolutionary Impact of Protein Flexibility
Source: PLoS One. 2013 Aug 21;8(8):e72225. doi: 10.1371/journal.pone.0072225 (PMC3749098; doi:10.1371/journal.pone.0072225)
Supplement: Table S3 — Dipeptide sequences enriched in ancient domains. Dipeptides are identified by participating amino acids using one-letter codes and listed together with statistical significance values of enrichment (p) and subsets specified by intervening amino acids corresponding to Group 1, 2 and 3 domain structures (one of 9 subsets possible). (PDF) [file pone.0072225.s011.pdf]

Table S3. Dipeptides that are enriched in ancient domains. Dipeptides are identified by participating amino acids using one-letter codes and listed together with statistical significance values of enrichment (p). The nine possible dipeptide subsets are specified by intervening amino acids corresponding to *Group 1*, *2* and *3* domain structures.

	Enrichment (p)	Subsets
AL	1.80E-10	21
LA	0.0000031	12
IA	0.0000033	22
GI	0.0000074	32
AR	0.000025	23
VV	0.000046	22
EA	0.000073	32
II	0.000096	22
VG	0.000098	23
RA	0.00012	32
VA	0.00019	22
GV	0.00031	32
IV	0.00031	22
LK	0.00036	12
AG	0.00044	23
RE	0.00047	33
LG	0.00073	13
AK	0.00079	22
GL	0.0009	31
EV	0.001	32
GK	0.001	32
MP	0.0013	22
AI	0.0018	22
PL	0.0021	21
TG	0.0022	23
DV	0.0027	32
AA	0.0039	22
IG	0.004	23
YA	0.0042	11
VI	0.0051	22
AH	0.0053	23
LV	0.0079	12
LR	0.009	13
VE	0.01	23
PI	0.011	22
AD	0.012	23
EG	0.019	33
AE	0.02	23
RY	0.021	31
KA	0.022	22
QA	0.022	32
EK	0.023	32
AV	0.025	22
EP	0.025	32
PG	0.025	23
VL	0.026	22
YG	0.026	13
PE	0.027	23
HI	0.028	32
GE	0.031	33
IR	0.032	23
GM	0.033	32
VP	0.036	22
KP	0.04	22
PT	0.041	22
ER	0.042	33
